# Toll-like receptor ligands sensitize B-cell receptor signalling by reducing actin-dependent spatial confinement of the receptor

**DOI:** 10.1038/ncomms7168

**Published:** 2015-02-03

**Authors:** Spencer A. Freeman, Valentin Jaumouillé, Kate Choi, Brian E. Hsu, Harikesh S. Wong, Libin Abraham, Marcia L. Graves, Daniel Coombs, Calvin D. Roskelley, Raibatak Das, Sergio Grinstein, Michael R. Gold

**Affiliations:** 1 Department of Microbiology & Immunology, University of British Columbia, 2350 Health Sciences Mall, Vancouver, British Columbia, Canada V6T 1Z3; 2 Department of Cellular & Physiological Sciences, University of British Columbia, 2350 Health Sciences Mall, Vancouver, British Columbia, Canada V6T 1Z3; 3 Life Sciences Institute I^3^ and Cell Research Groups, University of British Columbia, 2350 Health Sciences Mall, Vancouver, British Columbia, Canada V6T 1Z3; 4 Program in Cell Biology, The Hospital for Sick Kids Research Institute, 686 Bay Street, Toronto, Ontario, Canada M5G 0A4; 5 Department of Mathematics and Institute of Applied Mathematics, 1984 Mathematics Road, University of British Columbia, Vancouver, British Columbia, Canada V6T 1Z2; 6 Department of Integrative Biology, University of Colorado Denver, 1151 Arapahoe, Denver, Colorado 80204, USA

## Abstract

Integrating signals from multiple receptors allows cells to interpret the physiological context in which a signal is received. Here we describe a mechanism for receptor crosstalk in which receptor-induced increases in actin dynamics lower the threshold for signalling by another receptor. We show that the Toll-like receptor ligands lipopolysaccharide and CpG DNA, which are conserved microbial molecules, enhance signalling by the B-cell antigen receptor (BCR) by activating the actin-severing protein cofilin. Single-particle tracking reveals that increased severing of actin filaments reduces the spatial confinement of the BCR within the plasma membrane and increases BCR mobility. This allows more frequent collisions between BCRs and greater signalling in response to low densities of membrane-bound antigen. These findings implicate actin dynamics as a means of tuning receptor signalling and as a mechanism by which B cells distinguish inert antigens from those that are accompanied by indicators of microbial infection.

## Abstract (web summary)

Microbial pathogens can activate both innate and adaptive receptors, and integration of these signals may enhance the sensitivity of the immune response. Freeman *et al*. show that innate microbial cues sensitize B cells to antigen by increasing actin dynamics and reducing the actin-dependent confinement of the B-cell receptor.

## Graphical abstract

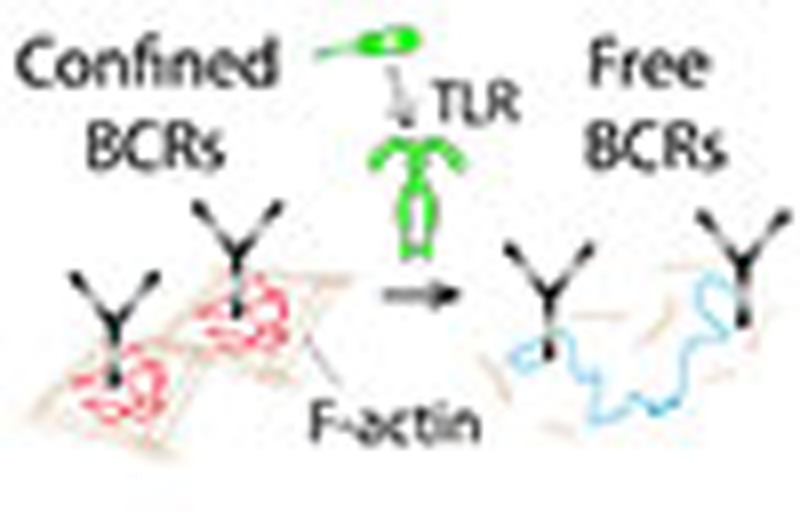

Cells routinely integrate signals from multiple receptors. Signals from one receptor can alter the threshold for cellular responses by modulating the surface expression or signalling output of another receptor, or alter the nature of the response by modulating downstream signalling pathways. Although the spatial organization of receptors and their mobility within the plasma membrane impact receptor signalling^1^, it is not clear whether receptor crosstalk can be mediated by changes in these parameters. Because B-cell receptor (BCR) mobility within the plasma membrane is a critical determinant of BCR signalling output^2^,^3^, we hypothesized that other receptors that impact B-cell activation modulate BCR mobility.

Differentiation of B-lymphocytes into antibody-producing cells is initiated by the antigen-specific BCR. However, the magnitude of the antibody response and the amount of antigen required to stimulate a response are determined by Toll-like receptors (TLRs), which recognize conserved microbial molecules^4^. TLR ligands include microbial components, for example, lipopolysaccharide (LPS) and DNA containing unmethylated CpG motifs^5^. Antigens associated with TLR ligands are effective immunogens, whereas non-infectious antigens elicit weak responses unless co-injected with adjuvants containing TLR ligands. The physical nature of the antigen determines whether B-cell-intrinsic TLR signalling is required for antibody responses. For soluble antigens, TLR ligands increase the ability of dendritic cells to activate T cells, which provide additional antigen-independent signals that facilitate B-cell activation^6^. In contrast, antibody responses to particulate antigens (for example, virus-like particles) require B-cell-intrinsic TLR signalling via the MyD88 adaptor protein^6^,^7^. *In vivo*, B cells are also activated by antigen-presenting cells (APCs) that capture antigens via Fc receptors and complement receptors^8^,^9^,^10^. Such two-dimensional arrays of membrane-bound antigens can cluster BCRs and initiate BCR signalling^8^,^11^. It is not known whether TLR ligands enhance B-cell responses to APC-bound antigens.

On contact with antigens that are mobile within a two-dimensional membrane, BCRs coalesce into microclusters that interact with CD19 and recruit phosphoinositide 3-kinase, Vav, and phospholipase C-γ2 (refs 12, 13, 14, 15). Forming these microsignalosomes^15^ increases the efficiency of BCR signalling and reduces the amount of antigen required for B-cell activation^13^,^16^,^17^. B-cell responses to membrane-associated antigens require CD19 and the CD19-associated protein CD81, which are both dispensable for responses to soluble antigens^13^,^18^. Thus, dynamic spatial reorganization of the BCR, and its interactions with other cell surface proteins, are unique determinants of B-cell responsiveness to membrane-associated antigens.

BCR mobility and clustering is controlled by the submembrane actin cytoskeleton^3^,^19^,^20^. In resting B cells, the BCR is kept relatively immobile by the cortical actin network, which is linked to the plasma membrane by ezrin^2^,^20^. This limits antigen-independent BCR clustering, allowing only low-level tonic signalling^2^ that is essential for B-cell survival^21^. Disrupting the actin meshwork removes barriers to BCR diffusion and is sufficient to induce BCR signalling^2^,^18^,^20^. Consistent with this, optimal BCR signalling in response to membrane-associated antigens requires two pathways that alter the actin cytoskeleton so as to allow increased BCR mobility and microcluster formation. BCR-induced inactivation of ezrin causes local release of the submembrane actin cytoskeleton from the membrane^20^. Concomitantly, activation of the Rap GTPase by the BCR promotes the severing of actin filaments by cofilin^22^,^23^.

The role of the actin cytoskeleton in controlling BCR mobility suggests that other receptors on B cells could tune the threshold for BCR signalling and B-cell activation by regulating actin dynamics. We show here that TLR signalling primes B cells by reducing the amount of antigen that is required for initiating BCR signalling. This receptor crosstalk is dependent on TLR-induced increases in BCR mobility that require actin severing by cofilin.

## Results

### TLR priming enhances BCR signalling at low antigen density

**Figure 1:**
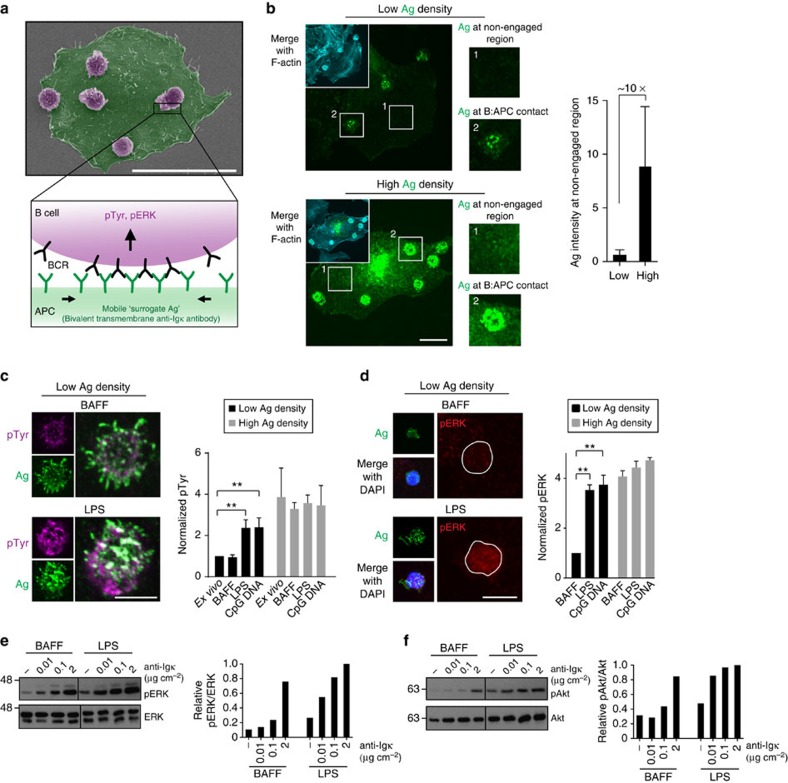
TLR priming increases the sensitivity of B cells to membrane-bound antigens. (**a**) Pseudocolored scanning EM image of B cells (purple) adhering to an APC (green) expressing a transmembrane rat anti-mouse Igκ antibody (surrogate antigen (Ag)). Scale bar, 50 μm. (**b**) Confocal images of *ex vivo* B cells that were added to APCs for 3 min before staining with Alexa488-anti-rat IgG to detect the surrogate antigen. Scale bar, 20 μm. Cells were visualized by F-actin staining (inset). To categorize APCs as having low or high antigen density, the gain was increased and the intensity of Alexa488-anti-rat IgG staining in regions of the APC that were not engaged by B cells was quantified (mean±s.e.m; *n*=30 cells from three experiments). (**c**,**d**) *Ex vivo* B cells, or B cells that had been cultured for 16 h with 5 ng ml^−1^ BAFF, BAFF+5 μg ml^−1^ LPS or BAFF+0.5 μg ml^−1^ CpG DNA, were added to APCs for 3 min and stained for surrogate antigen (Ag) and either pTyr (**c**) or pERK (**d**). At the settings used, only clustered antigen is detected. Confocal slices of pTyr staining at the contact site between B cells and APCs expressing low amounts of surrogate antigen (**c**). Staining for pERK, antigen and nuclei (4′,6-diamidino-2-phenylindole (DAPI) staining; outlined on pERK images) is shown as three-dimensional projections reconstructed from confocal slices (**d**). Scale bars, 5 μm. pTyr intensity at the B cell:APC contact site, as well as total pERK intensity, were quantified from images and normalized to those in BAFF-cultured B cells that had bound to APCs with a low density of surrogate antigen (mean±s.e.m. for three experiments; *n*>30 B cells per point). (**e**,**f**) B cells that had been cultured overnight with BAFF or BAFF+LPS were incubated for 10 min in wells coated with the indicated densities of anti-Igκ or 2 μg cm^−1^ anti-MHCII (−). pERK and total ERK (**e**) or pAkt and total Akt (**f**) blots are shown. pERK/ERK and pAkt/Akt ratios, normalized to those for LPS-cultured cells plated on 2 μg cm^−2^ anti-Igκ, are graphed (means for greater than three experiments). **P<0.01 using Student’s two-tailed unpaired *t*-test.

To test whether TLR ligands increase the sensitivity of B cells to membrane-associated antigens, B cells were added to APCs that had different cell surface densities of a transmembrane anti-Igκ light-chain antibody that can bind the BCR and act as a surrogate antigen^22^ (Fig. 1a,b). Within 3 min, clustering of this surrogate antigen, as well as phosphotyrosine (pTyr)-based BCR signalling, was observed at the B cell:APC contact site (Fig. 1b,c). When the surrogate antigen was present at low densities on the APC, B cells that had been cultured overnight with LPS or CpG DNA, ligands for TLR4 and TLR9 respectively, exhibited significantly greater pTyr signalling at the B cell:APC contact site (Fig. 1c) than resting B cells (*ex vivo* B cells or B cells that cultured overnight with the survival cytokine B-cell activation factor (BAFF)). They also exhibited higher levels of phosphorylated ERK (pERK) in the nucleus (Fig. 1d). In contrast, when B cells bound to APCs with high surrogate antigen density (~10-fold higher than APCs with low antigen density (Fig. 1b)), similar levels of pTyr and pERK signalling were observed in resting and TLR-activated B cells (Fig. 1c,d). Exposure to LPS also enhanced BCR-induced phosphorylation of ERK and Akt in response to low densities of anti-Igκ antibodies that were immobilized on plastic, such that pERK and pAkt levels induced by 0.1 μg cm^−2^ anti-Igκ in LPS-cultured cells were similar to those induced by 2 μg cm^−2^ in BAFF-cultured cells (Fig. 1e,f). This LPS-induced sensitization of BCR signalling occurred over a biologically relevant range of anti-Igκ densities. In BAFF-cultured B cells, 0.1 μg cm^−2^ immobilized anti-Igκ did not increase expression of the CD69 and CD86 activation markers, whereas maximal upregulation was induced by 2 μg cm^−2^ immobilized anti-Igκ (Supplementary Fig. 1). Thus, for two dimensional antigen arrays, TLR priming enhances BCR signalling when antigens are present at low, sub-optimal densities.

### TLR signalling increases BCR diffusion

The ability of B cells to initiate microcluster-based BCR signalling in response to two-dimensional antigen arrays depends on the BCR being mobile within the cell membrane^24^. Especially at low antigen density, the mobility of BCRs at the contact site may determine how many BCRs encounter antigen, cluster with other BCRs and initiate signalling. Because TLR priming enhanced BCR signalling induced by APC-bound and immobilized anti-Ig, we hypothesized that TLR ligands increased the mobility of the BCR within the plasma membrane.

To test this, BCR mobility was assessed via single-particle tracking (SPT), as done previously^2^,^25^. To visualize individual BCRs containing the membrane form of IgM (mIgM) as their antigen-binding subunit, B cells were labelled on ice with a limiting amount (1 ng ml^−1^) of monovalent biotin-conjugated anti-IgM Fab fragments, followed by streptavidin-conjugated Quantum dots (Qdots). Excess biotin was then added to prevent BCR crosslinking. The cells were adhered to coverslips coated with a non-stimulatory anti-MHCII antibody^2^,^18^ (Supplementary Fig. 2) and imaged in real time. Video recordings were analysed using u-track particle-tracking algorithms^26^ to reconstruct trajectories of individual mIgM molecules. We confirmed that our protocol labelled primarily single BCRs that were on the cell surface (Supplementary Fig. 3). The positional accuracy was calculated from the width of the Gaussian curve that was fit to the particle intensity profile obtained with our imaging system. This estimated error in determining particle positions was ~10 nm (Supplementary Fig. 4).

**Figure 2:**
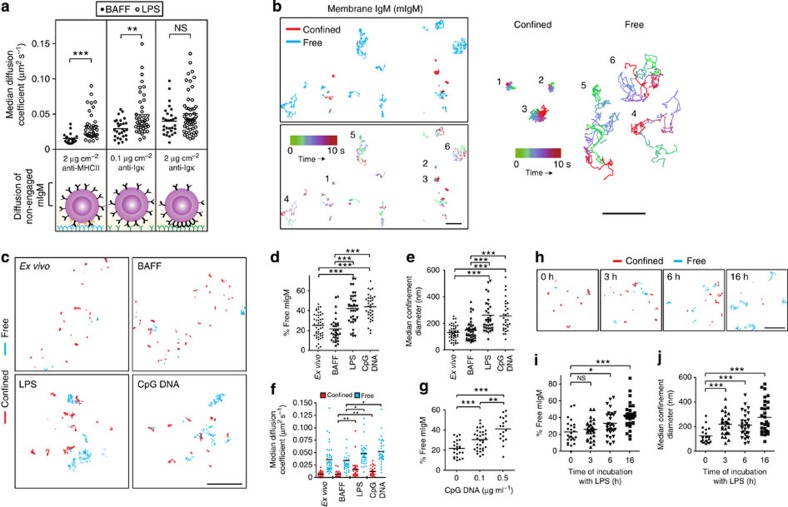
TLR ligands increase BCR mobility and decrease BCR confinement. (**a**) B cells cultured overnight with 5 ng ml^−1^ BAFF or BAFF+5 μg ml^−1^ LPS were labelled with biotinylated anti-IgM Fabs plus streptavidin Qdots, and then adhered for 5 min to coverslips coated with anti-MHCII or anti-Igκ. SPT of non-engaged mIgM-containing BCRs on the dorsal surface of cells (the ventral surface was imaged in all other SPT experiments) was performed. BCR trajectories generated from 10-s long 33 frames s^−1^ (Hz) videos were used to calculate single-state diffusion coefficients. Median values for individual B cells (>20 cells from three experiments) are plotted. Horizontal lines are mean values. See Supplementary Fig. 13 for MSS determination of free versus confined BCRs. (**b**) B cells were cultured overnight with BAFF+5 μg ml^−1^ LPS. Trajectories of mIgM-containing BCRs generated from 10-s 33 Hz videos were classified as confined or free. The same trajectories are shown with color-coded time intervals (enlarged in right panel). Scale bar, 1 μm. (**c**–**f**) SPT of BCRs on *ex vivo* B cells or B cells cultured overnight with 5 ng ml^−1^ BAFF, BAFF+5 μg ml^−1^ LPS or BAFF+0.5 μg ml^−1^ CpG DNA. BCR trajectories (**c**) generated from 10-s 33 Hz videos (see Supplementary Movies 1–3). Scale bar, 5 μm. MSS determination of free versus confined BCRs (**c**,**d**), median confinement diameters (**e**) and median diffusion coefficients for confined and free BCRs (**f**). Each dot is the median for trajectories from a single video. Horizontal lines are means for >30 videos; >1,500 trajectories analysed per condition. Similar results were obtained using Cy3-anti-IgM Fabs (see Supplementary Fig. 15). (**g**) The CpG DNA-induced increase in freely diffusing BCRs is dose dependent. (**h**–**j**) B cells were cultured overnight in BAFF, with 5 μg ml^−1^ LPS added for the last 0–16 h. Trajectories were generated from 10-s 33 Hz videos (**h**). Scale bar, 3 μm. Per cent of freely diffusing mIgM BCRs (**i**) and median confinement diameters (**j**). Each dot represents one video; >20 videos and >500 trajectories per condition. ***P<0.001, **P<0.01, *P<0.05, or NS (not significant) using Student’s 2-tailed unpaired *t*-test.

SPT was performed on primary B cells that were allowed to adhere for 5 min to coverslips coated with either anti-MHCII (to assess BCR mobility before antigen encounter) or anti-Igκ. Adhering B cells to immobilized anti-Igκ increased the diffusion coefficient (*D*) of non-engaged BCRs on the dorsal side of the cell in a dose-dependent manner (Fig. 2a), a relationship previously observed in A20 B-lymphoma cells^20^. Importantly, culturing B cells overnight with LPS increased the mobility of mIgM-containing BCRs, compared with those on BAFF-cultured B cells. This effect was observed when B cells were plated on anti-MHCII or on a low density of anti-Igκ (0.1 μg cm^−2^) but was not significant when cells were plated on high densities of anti-Igκ (2 μg cm^−2^). Thus, TLR signalling increases BCR mobility only when BCR signalling is suboptimal, presumably because BCR and TLR signalling converge on the same pathways that increase BCR mobility.

To gain further insights into how TLR priming alters BCR mobility, we imaged BCRs on the ventral side of the cell to obtain a larger number of trajectories, and then used multiple modelling approaches to analyse SPT data. Previous studies have characterized the mobility of individual BCRs by calculating a single diffusion coefficient for the period of observation^2^,^18^,^20^,^27^,^28^. Applying a similar ‘single-state’ analysis^29^ to our SPT data showed that the median diffusion coefficient for BCRs was greater in LPS-cultured cells than in resting B cells (Supplementary Fig. 5a,b).

Single-state analysis of receptor mobility is an oversimplification, as the mobility of proteins within the plasma membrane reflects dynamic interactions with barriers formed by the submembrane cytoskeleton and with membrane compartments that have distinct composition and viscosity (for example, lipid rafts, protein islands)^30^. Hence, individual receptor trajectories over a period of time consist of segments with different diffusion coefficients^29^. Therefore, we analysed BCR trajectories using a two-state hidden Markov model (HMM) that divides individual trajectories into slow- and fast-moving segments, and computes the rates of switching between the slow- and fast-moving states^29^. For the trajectories shown in Fig. 2h, this HMM analysis showed that exposing B cells to LPS for 16 h increased the diffusion coefficient for the slow state (*D*_slow_) by 2.68-fold relative to cells cultured overnight in BAFF (that is, 0 h LPS), and increased the diffusion coefficient for the fast state (*D*_fast_) by 1.65-fold (Supplementary Table 1). Importantly, LPS treatment altered the switching behaviour of the BCR so as to increase the ratio of slow-to-fast transitions versus fast-to-slow transitions (*K*_eff_). For the LPS-treated cells in Fig. 2h, slow-to-fast was the predominant mode of switching with *K*_eff_ being 1.21 (Supplementary Table 1). In contrast, *K*_eff_ for BAFF-cultured cells was 0.77, indicating that fast-to-slow transitions were more frequent in resting B cells. Thus, TLR ligands increase BCR mobility by promoting more frequent switching of the receptor to a fast-moving state.

### TLR signalling reduces BCR confinement

The ability of BCRs to encounter antigens on the surface of an APC is limited by the area that individual BCRs scan over a period of time (*t*). This area is proportional to the mean square displacement (MSD; *r*^2^) and depends on the receptor’s diffusion coefficient. For freely diffusing particles *r*^2^=4*D*Δ*t*. However, most receptors do not exhibit free diffusion due to the non-uniform nature of the plasma membrane as well as transient protein–protein and protein–lipid interactions. Studies of receptor trajectories indicate that their motion can be classified as free diffusion (MSD increases linearly over time), subdiffusive (the slope of MSD versus time decreases over time, a behaviour interpreted as being due to confinement), or super-diffusive (faster than free diffusion due to directed motion)^31^. Examining the relationship between particle displacement (*r*) and time when both quantities are raised to successively higher powers yields the moment scaling spectrum (MSS), which amplifies small differences from linearity and more effectively distinguishes the different modes of diffusion than MSD versus time^32^,^33^,^34^,^35^,^36^. Applying MSS analysis, as done by Jaqaman *et al*.^34^, allowed us to classify individual BCR trajectories as free or confined (subdiffusive) and to calculate their diffusion coefficients. Fewer than 5% of the trajectories exhibited directed or unclassified motion; these were not considered further. For receptors classified as confined, the maximal area explored by the receptor over the period of observation was used to determine the diameter of the confinement region. Figure 2b shows the displacement over time for BCRs within a single video and illustrates the MSS-based classification of trajectories as either free or confined. Dividing individual BCR trajectories into short time interval segments shows that a single track can contain both slow (shorter) and fast (longer) segments, consistent with the HMM analysis. Over a longer time interval (10–20 s), the area explored by BCRs that are classified as freely diffusing increases continually over time, whereas BCRs classified as confined/subdiffusive explore a limited area that does not increase steadily over time. The paths travelled by subdiffusive BCRs often double back such that the same area is explored repeatedly, suggesting that their mobility is confined in some manner.

MSS analysis showed that >75% of the BCRs on *ex vivo* and BAFF-cultured B cells exhibited confined trajectories (Fig. 2c,d and Supplementary Movie 1), with median confinement diameters of 120–160 nm (Fig. 2e, Supplementary Fig. 6a, Supplementary Table 2) and diffusion coefficients that were much lower than those of freely diffusing BCRs (Fig. 2f). Culturing B cells overnight with TLR ligands enhanced BCR mobility in three ways: it increased the fraction of freely diffusing BCRs by approximately twofold (Fig. 2d,g,i; compare Supplementary Movie 1 with Supplementary Movies 2 and 3), caused a twofold increase in the median diameter of the confinement area for BCRs exhibiting confined/subdiffusive motion (Fig. 2e,j, Supplementary Fig. 6a, and Supplementary Table 2), and increased the diffusion coefficients for both the confined and free receptors (Fig. 2f). This last effect is consistent with the increased switching of BCRs to a fast-moving state, as revealed by the HMM analysis. The increase in BCR mobility caused by TLR ligands was dose dependent (Fig. 2g) and required 3–6 h (Fig. 2h–j). Together the complementary HMM and MSS particle-tracking algorithms show that TLR signalling increases the probability that a BCR will transition to a fast-moving state that is either unconfined, or within a confinement zone that is larger than those in resting B cells.

### The actin network controls BCR diffusion and confinement

**Figure 3:**
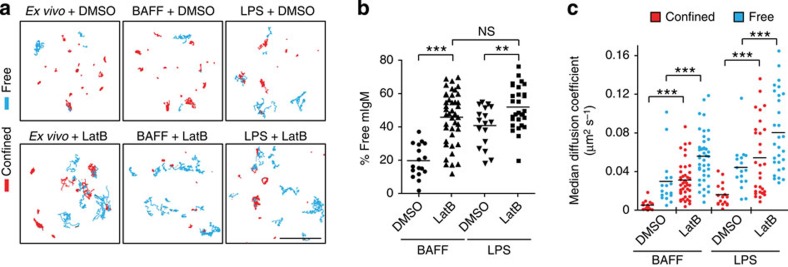
BCR confinement and diffusion are controlled by the actin cytoskeleton. *Ex vivo* B cells, as well as B cells that had been cultured overnight with either 5 ng ml^−1^ BAFF or BAFF+5 μg ml^−1^ LPS, were treated with dimethylsulphoxide (DMSO) or 1 μM latrunculin B (LatB) for 3 min before performing SPT as in Fig. 2. (**a**) BCR trajectories were generated from 10-s 33 Hz videos (compare Supplementary Movies 4 and 5 to Supplementary Movies 1 and 2). Scale bar, 5 μm. Red, confined trajectories; cyan, free trajectories. (**b**,**c**) The per cent of mIgM BCRs that exhibited free diffusion is shown (**b**), along with the median diffusion coefficients for both confined and free mIgM-containing BCRs (**c**). Horizontal lines represent the mean values for >15 videos per condition; >300 trajectories per condition were analysed. ***P<0.001, **P<0.01, or NS (not significant) using Student’s 2-tailed unpaired *t*-test.

Consistent with previous studies^2^, BCR mobility, as assessed by single-state diffusion coefficients, increased when resting B cells were treated with latrunculin for 3 min to partially disrupt the actin cytoskeleton (Supplementary Fig. 5b). HMM analysis revealed that treating BAFF-cultured B cells with latrunculin increased the diffusion coefficients for both the slow- and fast-moving states, and reduced the rate of fast-to-slow switching such that *K*_eff_ increased from 0.66 to 1.27 (Supplementary Table 1). MSS analysis showed that disrupting the actin cytoskeleton with latrunculin caused an approximately twofold increase in the fraction of BCRs exhibiting free diffusion, such that it was similar to that in LPS-treated cells (Fig. 3a,b and Supplementary Movie 4). For BCRs classified as confined/subdiffusive, latrunculin treatment caused a twofold increase in the confinement area (Supplementary Table 2). It also increased the median diffusion coefficients for both confined and free BCRs (Fig. 3c), consistent with the single-state and HMM analyses. This suggests that the actin cytoskeleton limits BCR mobility not only by creating barriers that confine receptors but also by controlling local diffusion rates, even in regions of the membrane in which receptors are not confined, as defined by the MSS algorithm^34^. This was true even for LPS-activated cells, where latrunculin treatment caused further increases in diffusion coefficients (Fig. 3c, Supplementary Table 1), confinement diameters (Supplementary Table 2), and the fraction of BCRs exhibiting free diffusion (Fig. 3b, Supplementary Movie 5).

### TLR ligands increase actin dynamics and cofilin activity

**Figure 4:**
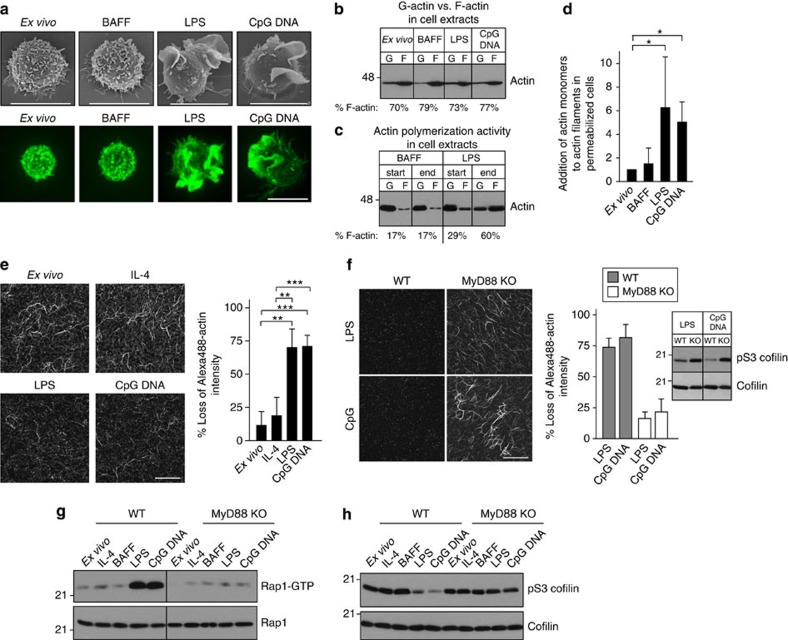
TLR signalling enhances actin dynamics and activates cofilin. B cells were analysed *ex vivo* or after overnight culture with survival cytokines (5 ng ml^−1^ BAFF or IL-4), with or without TLR ligands (5 μg ml^−1^ LPS or 0.5 μg ml^−1^ CpG DNA). (**a**) Scanning EM (*top*); *z* projections generated from *xy* confocal images of Alexa488-phalloidin-stained cells (bottom). Scale bars, 10 μm. (**b**) Cell extracts were separated into soluble and insoluble fractions containing G- and F-actin, respectively, and probed for actin. (**c**) Cell extracts were sonicated to destroy existing actin filaments and kept on ice (start) or incubated for 10 min at 37 °C to allow *de novo in vitro* actin polymerization (end). Samples were then separated into soluble and insoluble fractions to monitor the conversion of G-actin to F-actin. **b**,**c** show representative blots from three independent experiments. For each sample, the per cent of total actin present as F-actin is indicated below each pair of lanes. (**d**) Cells were permeabilized in the presence of Alexa488-actin monomers for 10 s. Incorporation of labelled monomers into actin filaments was quantified by determining the Alexa488-actin/F-actin ratio for >30 cells per condition. The values are normalized to *ex vivo* cells (mean±s.e.m.; three experiments). (**e**,**f**) *In vitro* severing of immobilized fluorescent F-actin filaments by cell extracts. F-actin severing was quantified by imaging before and after adding cell extracts, and then determining the average loss of fluorescence for multiple fields (mean±s.e.m.; four experiments). The blot in **f** shows inactive cofilin that is phosphorylated on S3 (pS3 cofilin) as well as total cofilin in the same cell extracts that were used for the severing assay. (**g**,**h**) Levels of activated Rap1-GTP and total Rap1 (**g**) or pS3 cofilin and total cofilin (**h**) in extracts of *ex vivo* B cells or B cells that were cultured overnight with the indicated cytokines or TLR ligands. Similar results were obtained in 5 experiments. ***P<0.001, **P<0.01, *P<0.05 using Student’s two-tailed unpaired *t*-test.

Because TLR priming had similar effects on BCR diffusion and confinement as latrunculin, we asked whether TLR signalling increased actin dynamics. Increased turnover of the submembrane actin cytoskeleton would reduce the lifetime and local density of actin barriers that limit the diffusion of membrane proteins, mimicking the effects of latrunculin. Culturing B cells with LPS or CpG DNA caused a marked reorganization of the actin cytoskeleton characterized by the formation of F-actin-rich membrane ruffles (Fig. 4a). Although TLR stimulation did not alter the ratio of F-actin to G-actin (Fig. 4b), TLR-activated B cells had greater actin-polymerizing activity than resting B cells. This was shown by disassembling pre-existing F-actin polymers in cell extracts by sonication, and then measuring the *de novo in vitro* polymerization of actin into insoluble filaments (Fig. 4c). Moreover, when added to semipermeabilized cells, more fluorescent actin monomers were incorporated into actin filaments in TLR-activated B cells than in resting B cells (Fig. 4d). This suggests that there was an increase in the number of barbed ends, sites of actin monomer addition that are formed when F-actin is severed. Indeed, TLR-activated B cells had increased F-actin severing activity compared to resting B cells, as assessed by the ability of cell extracts to cleave fluorescent F-actin filaments *in vitro* (Fig. 4e,f). The LPS- and CpG DNA-induced increase in actin-severing activity was not observed in B cells from mice lacking the MyD88 adaptor protein (Fig. 4f) that links TLRs to downstream signalling pathways^5^. Thus, TLR signalling increases both actin-severing and actin-polymerizing activities in B cells. This suggests that the F-actin networks in TLR-primed B cells are more dynamic, being rapidly turned over and reassembled such that local barriers to receptor diffusion are more transient.

The Rac1, RhoA and Rap1 GTPases control actin organization and dynamics. Consistent with a recent report^37^, the level of active GTP-bound Rac1, which promotes actin polymerization and reorganization, was slightly elevated in LPS-treated cells compared with BAFF-cultured cells (Supplementary Fig. 7a). Conversely, the amount of active RhoA, which stabilizes actin filaments and promotes formin-mediated actin polymerization, was slightly decreased (Supplementary Fig. 7b). In contrast to these modest effects, TLR signalling via MyD88 markedly increased the levels of active GTP-bound Rap1 (Fig. 4g).

In B cells, Rap1-GTP increases actin dynamics via cofilin^22^, which is the major actin-severing protein in LPS-activated B cells^22^. Actin-severing proteins increase actin dynamics by coupling severing to polymerization^38^. Filament severing increases the pool of actin monomers while generating barbed ends where the Arp2/3 complex can nucleate the formation of new actin filaments^39^. Because TLR stimulation increased both actin-severing activity and actin polymerization in B cells, and also activated Rap1, we asked if cofilin was activated by TLR signalling. The ability of cofilin to bind actin filaments and initiate severing requires its dephosphorylation on serine 3 (S3) (ref. 40). Culturing B cells with LPS or CpG DNA resulted in substantial dephosphorylation of cofilin on S3, which was dependent on MyD88 (Fig. 4h). Although activated Rap1 also promotes integrin activation in B cells^41^, treating B cells with Mn^2+^ to directly activate integrins^42^ did not increase BCR mobility (Supplementary Fig. 8). Therefore, we asked whether TLR effects on BCR mobility were dependent on cofilin-mediated actin severing.

### TLR enhancement of BCR mobility depends on actin severing

**Figure 5:**
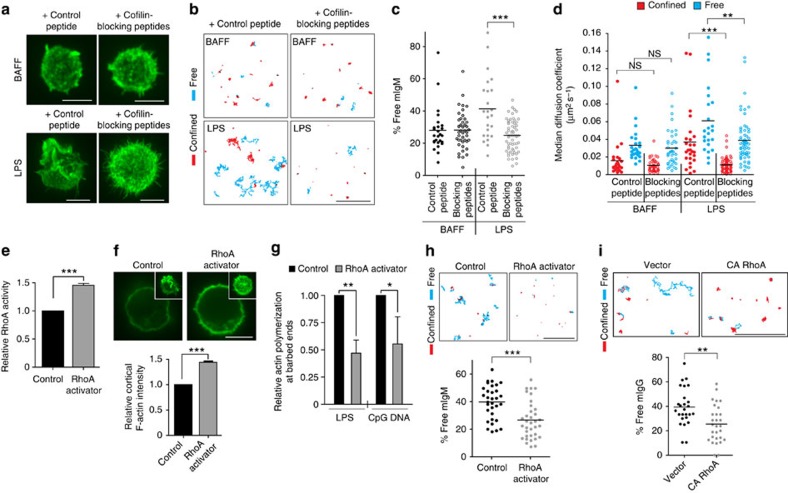
TLR enhancement of BCR mobility is dependent on actin severing. (**a**–**d**) B cells that had been cultured overnight with either 5 ng ml^−1^ BAFF or BAFF+5 μg ml^−1^ LPS were treated with 5 μM of the M/W cofilin-blocking peptides or the control Q peptide for 5 min at 37 °C. **a** shows *z* projections of F-actin staining. In **b**–**d**, BCR trajectories were generated from 10-s 33 Hz videos (see Supplementary Movie 6). Scale bar, 5 μm. Red, confined trajectories; cyan, free trajectories. The per cent of mIgM BCRs that exhibited free diffusion (**c**) and the median diffusion coefficients for confined and free mIgM-containing BCRs (**d**) are shown. Horizontal lines are mean values for >20 videos; >600 trajectories per condition were analysed. (**e**–**h**) B cells that were cultured overnight with BAFF+5 μg ml^−1^ LPS were incubated with or without the RhoA-activating peptide for 2–3 h. In **e**, a RhoA-specific G-LISA was used to quantify RhoA activity (normalized to untreated cells; mean±s.e.m. for three experiments). In **f**, cells were stained with Alexa488-phalloidin. Cortical F-actin intensity was measured at regions in the *xy* planes that excluded membrane ruffling. Fluorescence per unit area was normalized to control cells (mean±s.e.m.; three experiments). Insets show *z* projections. In **g**, cells were permeabilized and the addition of Alexa488-actin monomers at the barbed ends of actin filaments was quantified as in Fig. 4d. Actin polymerization is expressed relative to control cells (mean±s.e.m.; three experiments). **h** shows SPT of mIgM-containing BCRs (see Supplementary Movie 7) and the per cent of BCRs exhibiting free diffusion. (**i**) A20 cells were transfected with constitutively active RhoA (CA RhoA) or empty vector. SPT of the membrane IgG (mIgG)-containing BCRs on these cells was performed and the per cent of BCRs exhibiting free diffusion is graphed (mean±s.e.m.; three experiments). Scale bars in **b**,**h**,**i** are 3 μm. The mean positional accuracy for mIgG-containing BCRs that were visualized with biotinylated anti-IgG Fab fragments plus streptavidin-655 nm-Qdots was 18.9 nm (see Supplementary Fig. 4). ***P<0.001, **P<0.01, *P<0.05, or NS (not significant) using Student’s two-tailed unpaired *t*-test.

To block cofilin-mediated actin severing, we used a combination of two cell-permeable peptides (M, W)^43^, each consisting of penetratin coupled to one of the two F-actin-binding regions of cofilin. These peptides block the binding of cofilin to F-actin in T cells^43^ and blocked the *in vitro* severing of F-actin filaments by extracts of LPS-stimulated B cells (Supplementary Fig. 9). Moreover, treating LPS-activated B cells with the M/W peptides resulted in the elimination of membrane ruffles and a more homogeneous cortical F-actin density (Fig. 5a), indicating that blocking F-actin severing decreased actin dynamics. The control Q peptide-penetratin conjugate, in which residues required for actin binding were mutated^43^, had no effect on membrane ruffling or actin organization in LPS-activated B cells (Fig. 5a). Importantly, MSS analysis showed that treating LPS-activated B cells with the M/W cofilin-blocking peptides reduced the fraction of freely diffusing BCRs (Fig. 5b,c and Supplementary Movie 6), reduced the median diffusion coefficients for both confined and free BCRs (Fig. 5d), and reduced the confinement areas for confined/subdiffusive BCRs (Supplementary Table 2, Supplementary Fig. 6b). Similarly, single-state analysis showed that the M/W peptides reduced the median diffusion coefficient of BCRs relative to BCRs in LPS-activated B cells that had been treated with the Q peptide (Supplementary Fig. 5c). HMM analysis revealed the M/W peptides reduced *D*_slow_ and *D*_fast_ and increased the frequency of fast-to-slow transitions so as to reduce *K*_eff_ (Supplementary Table 1). Together these data indicate that promoting F-actin severing is a key mechanism by which TLR signalling reduces BCR confinement and enhances BCR mobility.

If TLR signalling enhances BCR mobility by promoting F-actin severing and increasing the turnover dynamics of the submembrane actin cytoskeleton, then increasing the density and stability of cortical F-actin should oppose TLR effects on BCR mobility. To test this, we treated TLR-activated B cells with cell-permeable *E. coli* cytotoxic necrotizing factor, which directly activates RhoA via deamidation of Gln63 (ref. 44). Activated RhoA stabilizes actin filaments and treating TLR-activated B cells with the RhoA activator mimicked the effects of the cofilin-blocking peptides, suppressing membrane ruffling, increasing the density of cortical F-actin, and decreasing actin polymerization at the barbed ends that are generated by filament severing (Fig. 5e–g). As determined by the MSS, HMM and single-state analyses, the RhoA activator decreased BCR mobility and increased BCR confinement (Fig. 5h, Supplementary Fig. 5d, Supplementary Fig. 6c, Supplementary Tables 1 and 2 and Supplementary Movie 7), similar to when the cofilin-blocking peptides were used to prevent F-actin severing. Treating LPS-activated B cells with the RhoA activator or with cofilin-blocking peptides also made the state-switching behaviour of the BCR resemble that of resting B cells in that fast-to-slow transitions were more frequent than slow-to-fast (*K*_eff_<1; Supplementary Table 1). To validate the use of the RhoA-activating peptide, we showed that expressing a constitutively active form of RhoA (CA RhoA) in the A20 B-cell line also decreased BCR mobility (Fig. 5i). These findings indicate that the ability of TLR signalling to reduce BCR confinement and increase BCR mobility is dependent on actin severing.

**Figure 6:**
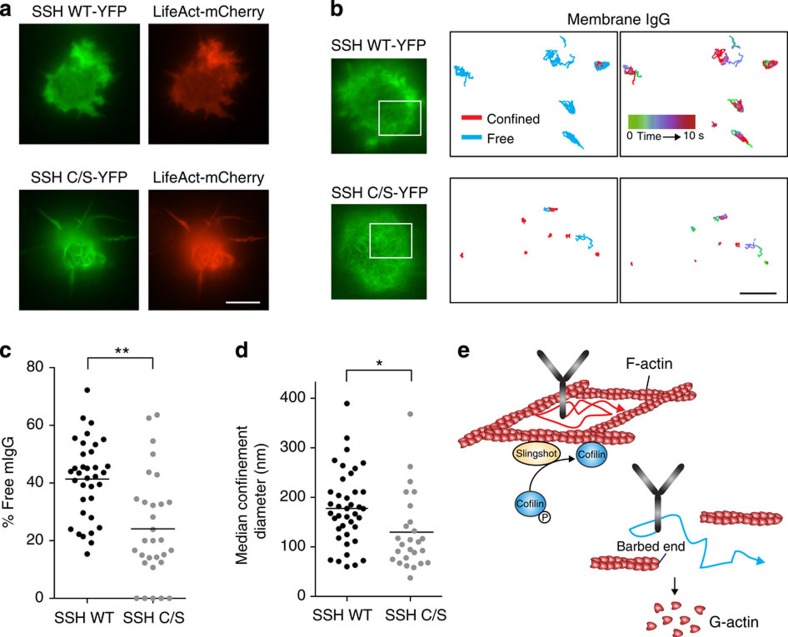
Cofilin activity controls BCR confinement. A20 cells were transiently co-transfected with plasmids encoding LifeAct-mCherry and either WT SSH or the dominant-negative catalytically inactive form of SSH (SSH C/S) fused to yellow fluorescent protein (YFP). (**a**) Epifluorescent images of dually transfected A20 cells plated on anti-MHCII-coated coverslips. Scale bar, 10 μm. The increased F-actin density (indicated by the LifeAct-mCherry fluorescence) in the SSH C/S-transfected cells is indicative of cofilin-mediated actin severing being inhibited. (**b**) For SSH WT-transfected cells (top panels) and SSH C/S-transfected cells (lower panels), SPT was performed for mIgG-containing BCRs in the regions indicated by the white boxes. BCR trajectories were generated from 10-s 33 Hz videos. Red, confined trajectories; cyan, free trajectories. The same trajectories are shown with color-coded time intervals. Scale bar, 1 μm. (**c**,**d**) The per cent of mIgG BCRs that exhibited free diffusion (**c**) and the median confinement diameter for confined mIgG-containing BCRs (**d**) are shown. Each dot is the median for trajectories from a single video (*n*>25 videos from three experiments). (**e**) Model showing how WT SSH promotes dephosphorylation of cofilin, which allows cofilin to bind F-actin and initiate filament severing. By severing actin filaments, activated cofilin removes barriers to BCR diffusion. For confined BCRs, this either increases confinement region size or allows free diffusion.

To further implicate cofilin-mediated actin severing in the regulation of BCR mobility, we modulated the ability of cofilin to bind F-actin, an interaction that requires dephosphorylation of S3 on cofilin. This reaction is mediated by the Slingshot (SSH) phosphatase^45^. A catalytically inactive form of SSH in which a cysteine residue in the active site is mutated to serine (SSH-C/S) acts in a dominant-negative manner with respect to SSH-mediated dephosphorylation of cofilin^45^. Expressing SSH-C/S in B cells increases the levels of phosphorylated (inactive) cofilin and blocks processes that are dependent on cofilin-mediated actin severing, including BCR microcluster formation^22^. To assess the role of cofilin activation and cofilin-mediated actin severing in regulating BCR mobility, we expressed wild-type SSH (SSH WT) or SSH-C/S in A20 cells along with LifeAct-mCherry (Fig. 6a). Cells expressing high levels of SSH-C/S exhibited dramatic bundling and buckling of actin filaments, consistent with impaired F-actin severing (Fig. 6a). We then used SPT to assess BCR mobility in A20 cells expressing low levels of SSH WT or SSH-C/S. Compared with cells expressing SSH WT, SSH-C/S expression increased BCR confinement, as indicated by a lower per cent of freely diffusing receptors and a decrease in the size of confinement zones (Fig. 6b–d). This suggests that SSH-mediated dephosphorylation and activation of cofilin is required to release the BCR from confinement zones (Fig. 6e).

### TLR ligands increase BCR collisions and tonic signalling

**Figure 7:**
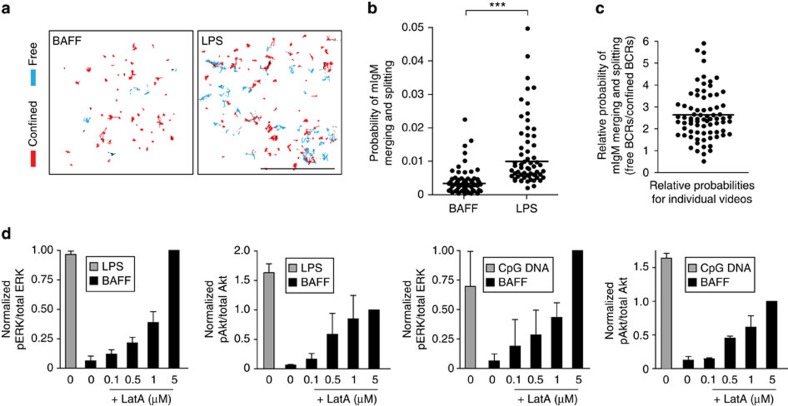
TLR ligands increase BCR–BCR collisions and tonic signalling. (**a**–**c**) SPT of mIgM-containing BCRs on B cells that had been cultured overnight with 5 ng ml^−1^ BAFF or BAFF+5 μg ml^−1^ LPS. Trajectories were generated from 5-s 10 Hz videos (**a**). Scale bar, 3 μm. Red, confined trajectories; cyan, free trajectories. The probability of a single BCR undergoing a collision with another BCR in a 5-s video was determined by applying merge-split algorithms to the trajectories (**b**). Each dot represents the probability for all of the BCRs in an individual video (*n*>50 videos from three experiments). The relative probability of free versus confined BCRs undergoing merge-split events in LPS-stimulated cells is shown in panel **c**. Each dot represents the relative probability for free versus confined BCRs in an individual video (*n*>75 videos from three experiments). (**d**) B cells were cultured overnight with 5 ng ml^−1^ BAFF, BAFF+5 μg ml^−1^ LPS, or BAFF+0.5 μg ml^−1^ CpG DNA and then treated with dimethylsulphoxide (DMSO (0)) or the indicated concentrations of latrunculin A (LatA) for 5 min. Cell extracts were analysed by immunoblotting with anti-phospho-ERK (pERK) or anti-phospho-Akt (pAkt) as well as total ERK or Akt. Graphs show the ratios of pERK/ERK signals or pAkt/Akt signals from at least two experiments (average±range or mean±s.e.m.). Representative blots are shown in Supplementary Fig. 10. ***P<0.001 using Student’s two-tailed unpaired *t*-test.

Tonic Ag-independent BCR signalling, which is required for B-cell survival^21^, is likely due to random BCR–BCR collisions as well as the interaction of BCR nanoclusters with CD19 (refs 18, 46). Because such physical interactions are limited by the actin cytoskeleton^18^,^46^, the enhanced actin dynamics and BCR mobility in TLR-activated B cells could increase BCR–BCR collisions and antigen-independent BCR signalling. To test this, we analysed SPT data using merge-split detection algorithms^34^ to quantify BCR–BCR collisions, which were defined as pairs of BCR trajectories that coalesced and then separated. We found that the probability of BCR collisions increased approximately threefold when B cells were cultured overnight with LPS (Fig. 7a,b). This may be attributable to the TLR-induced increase in freely diffusing BCRs, which had a greater probability of undergoing collisions than confined BCRs (Fig. 7c).

The increased frequency of BCR–BCR collisions in TLR-activated B cells correlated with increased levels of pERK and phospho-Akt (pAkt; Fig. 7d (compare left two bars on each graph) and Supplementary Fig. 10 (compare 0 LatA lanes)) in the absence of any BCR ligand. Clustering the BCR with anti-Ig antibodies induced further activation of ERK and Akt (see Fig. 1e,f and Supplementary Fig. 11). Interestingly, the ERK and Akt phosphorylation caused by TLR ligands could be ablated by briefly treating the cells with the Syk inhibitor piceatannol (Supplementary Fig. 12), consistent with this being antigen-independent BCR signalling. The idea that TLRs co-opt BCR signalling is supported by the observations that LPS-induced B-cell proliferation is significantly impaired in B cells lacking key BCR signalling components such as Btk^47^, CD19 (ref. 48), BLNK^49^ and Vav^50^. The TLR-induced increase in tonic signalling could be recapitulated by briefly treating BAFF-cultured cells with latrunculin to disrupt the actin cytoskeleton (Fig. 7d, Supplementary Fig. 10). Latrunculin-induced stimulation of early BCR signalling events (Ca^2+^ flux, phosphorylation of ERK and Akt) requires the presence of the BCR as well as the BCR signalling pathway components Lyn, Btk, CD19, phospholipase C-γ2, Vav and phosphoinositide 3-kinase^2^,^18^. Together these data suggest that TLR ligands induce antigen-independent BCR signalling by increasing actin dynamics and BCR mobility.

### TLR and BCR signalling converge on cofilin

**Figure 8:**
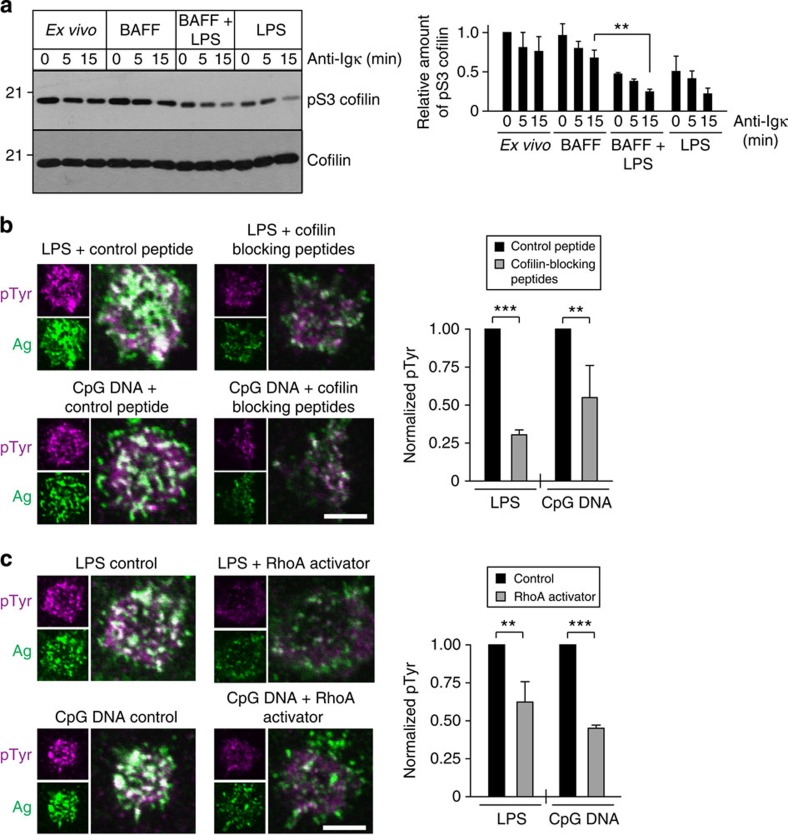
B-cell responses to APC-bound antigens are dependent on actin dynamics that correlate with cofilin activation. (**a**) *Ex vivo* B cells, as well as B cells that were cultured overnight with 5 ng ml^−1^ BAFF, BAFF+5 μg ml^−1^ LPS or LPS alone, were stimulated with 1 μg ml^−1^ anti-Igκ for the indicated times. The levels of pS3 cofilin and total cofilin in the cell extracts were assessed by immunoblotting (left panel). The pS3 cofilin/total cofilin ratio, relative to that in unstimulated *ex vivo* B cells, is graphed (mean±s.e.m. for three experiments). (**b**,**c**) B cells were cultured overnight with 5 ng ml^−1^ BAFF plus either 5 μg ml^−1^ LPS or 0.5 μg ml^−1^ CpG DNA. Before being added to APCs for 3 min, the B cells were treated with cofilin-blocking peptides or the control Q peptide (**b**), or incubated with or without the RhoA activator peptide (**c**), as in Fig. 5. B cells that were in contact with APCs expressing a low level of surrogate antigen were imaged. Representative images of pTyr and surrogate antigen staining at the B cell:APC contact site are shown. Scale bar, 5 μm. The pTyr signal at the contact site was quantified for >20 cells from three experiments (mean±s.e.m. relative to control cells). ***P<0.001; **P<0.01 using Student’s two-tailed unpaired *t*-test.

Exposing B cells to TLR ligands increased BCR signalling in response to low densities of immobilized and APC-bound antigen (Fig. 1). As shown in Fig. 2a and Supplementary Fig. 13, TLR priming and acute antigen-induced BCR signalling both increased BCR mobility, with additive increases when antigen was present at low density. Because both TLR priming (Fig. 4h) and short-term BCR engagement^22^ induce cofilin dephosphorylation, we asked whether TLR and BCR signalling converged on cofilin. Indeed, overnight exposure to LPS decreased cofilin phosphorylation in primary B cells, and a subsequent 5–15-min stimulation with anti-Igκ led to a further decrease in the amount of cofilin in the phosphorylated, inactive state (Fig. 8a). TLR priming plus BCR stimulation caused significantly greater cofilin dephosphorylation/activation than BCR stimulation alone in BAFF-cultured cells.

The combined effects of TLR and BCR signalling on cofilin activation and BCR mobility may augment the ability of B cells to respond to low densities of APC-bound antigen by increasing antigen–BCR encounters and the formation of BCR microclusters that assemble signalosomes. To test whether the initiation of BCR signalling by APCs expressing low amounts of surrogate antigen is dependent on cofilin-mediated actin severing, we pretreated B cells with the cofilin-blocking peptides, or with the RhoA activator, to increase the density and stability of the submembrane actin network that limits BCR mobility. Both these treatments significantly reduced the amount of pTyr-based signalling at the B cell–APC contact site (Fig. 8b,c). Thus, the combined ability of TLR priming and BCR signalling to activate cofilin and increase actin dynamics is important for APC-associated antigens to stimulate BCR signalling.

### Marginal zone B cells resemble TLR-primed B cells

**Figure 9:**
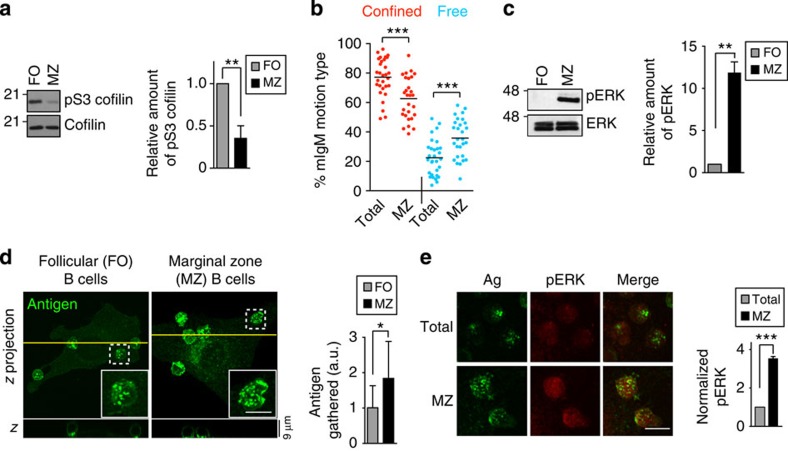
MZ B cells have greater cofilin activation, BCR mobility and BCR signalling than FO B cells. (**a**) Immunoblots showing the levels of pS3 cofilin and total cofilin in extracts of *ex vivo* FO and MZ B cells. In the graph, the ratio of pS3 cofilin/total cofilin is expressed relative to that in FO B cells (mean±s.e.m. for three experiments). (**b**) SPT of mIgM-containing BCRs on FO and MZ B cells. MSS analysis was used to determine the per cent of BCRs exhibiting confined versus free motion. Each dot is the median value for trajectories from a single video. Horizontal lines are mean values for >28 videos; >1,400 trajectories were analysed for each cell population. (**c**) Immunoblots showing the levels of pERK and total ERK in extracts of *ex vivo* FO or MZ B cells. In the graph, the ratio of pERK/ERK is expressed relative to that in FO B cells (mean±s.e.m. for three experiments). (**d**) FO and MZ B cells were added to APCs for 3 min before staining for the surrogate antigen. *z* projections (left panel, upper image*s*) are shown, along with *En face* views along the yellow line (lower images). The *en face* views show gathered antigen at the B cell:APC contact site (bottom of image). The insets show enlarged images of B cells that have adhered to the APC. Antigen gathered into microclusters was quantified in arbitrary units (a.u.) and is expressed relative to that in FO B cells. Mean±s.e.m. for >30 cells from two experiments. (**e**) Total splenic B cells (of which >90% are FO B cells) or MZ B cells were added to APCs for 3 min before staining for pERK and the surrogate antigen. *z* projections are shown. Total pERK signals were quantified and normalized to those in FO B cells. Mean±s.e.m. for three independent experiments with *n*>30 for each B-cell population. Note that MZ and FO B cells had nearly identical levels of BCR on the cell surface, as assessed by fluorescence-activated cell sorting analysis of cells stained with anti-Igκ-PerCP-5.5 (see Supplementary Fig. 16). ***P<0.001, **P<0.01, *P<0.05 using Student’s two-tailed unpaired *t*-test.

To assess whether modulating cofilin activity is a general mechanism for tuning B-cell responses to antigen, we compared follicular (FO) and marginal zone (MZ) B cells. MZ B cells are highly responsive to TLR stimulation and mount rapid antibody responses to microbial antigens when activated via combined BCR and TLR signalling^51^. MZ B cells have a ‘constitutive partially activated’ phenotype that resembles TLR-activated FO B cells^51^. Consistent with this, cofilin phosphorylation was lower in *ex vivo* MZ B cells than in *ex vivo* FO B cells (Fig. 9a). The greater amount of active dephosphorylated cofilin in MZ B cells correlated with a greater fraction of BCRs exhibiting free diffusion, as opposed to confined motion, compared to FO B cells (Fig. 9b). This increased BCR mobility was associated with ~10-fold higher basal levels of pERK (Fig. 9c). Importantly, on binding to the same population of surrogate antigen-expressing APCs, MZ B cells exhibited a greater ability to gather antigen into microclusters (Fig. 9d,e) than FO B cells and this correlated with threefold higher levels of pERK (Fig. 9e). Hence, MZ B cells resemble TLR-primed B cells in having greater amounts of active cofilin, increased BCR mobility, higher levels of tonic signalling and increased BCR signalling in response to APC-bound antigens.

## Discussion

**Figure 10:**
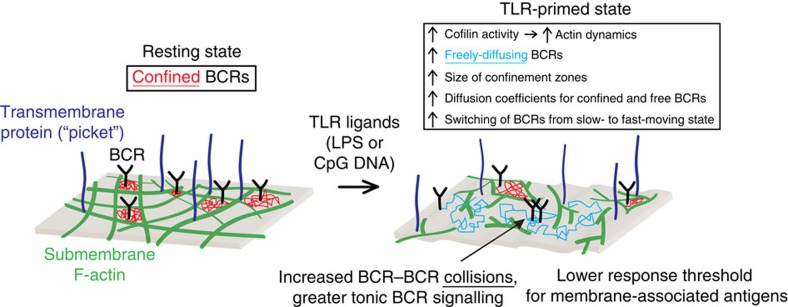
Model for TLR-induced transition of B cells to a primed state. The TLR-primed state is characterized by increased cofilin activity, which increases the turnover dynamics of the actin cytoskeleton. This reduces actin-based barriers to BCR diffusion, resulting in an increased number of BCRs exhibiting free diffusion, an increase in the size of confinement zones, increased diffusion coefficients for both free and confined BCRs and increased switching of BCRs from slow- to fast-moving states. The increased BCR mobility allows more frequent BCR–BCR collisions, which may lead to increased antigen-independent tonic BCR signalling. Increased BCR mobility would also enhance microcluster formation when APC-bound antigens are present at low densities, resulting in greater antigen-induced BCR signalling. In this way, TLR priming increases the sensitivity of B cells to low densities of membrane-bound antigens.

B-cell responses to membrane-bound antigens involve the formation of BCR microclusters that assemble microsignalosomes. This spatial reorganization of BCRs requires their release from confinement zones that are structured by F-actin and tetraspanins^2^,^18^,^20^. We have shown that TLR signalling increases actin turnover dynamics such that BCR confinement is reduced, the diffusion of both free and confined/subdiffusive BCRs is increased, and the switching of BCRs from slow- to fast-moving states is increased (Fig. 10). This enhanced BCR mobility increases antigen-independent BCR–BCR collisions as well as tonic signalling, resulting in a primed state in which the context provided by TLR ligands reduces the amount of antigen that is required to trigger maximal BCR signalling. Such a primed state may be recapitulated by MZ B cells, which are specialized for mounting rapid responses to microbial antigens^51^. Cooperation between TLR and BCR signalling is a key aspect of B-cell activation, and allows B cells to discriminate between microbial and inert antigens. Our findings reveal a new mechanism for how TLRs regulate BCR signalling and control the threshold for BCR activation.

Previous studies of BCR mobility have calculated single-state diffusion coefficients. To gain greater insights into BCR mobility and spatial confinement, we employed two additional modelling approaches. MSS analysis has been used to distinguish free diffusion from confined/subdiffusive motion^26^,^34^,^35^,^36^. Applying MSS analysis to SPT data showed that TLR signalling enhanced BCR mobility not only by increasing the proportion of freely diffusing receptors but also by increasing the size of confinement zones. Because the mode of diffusion exhibited by a single receptor may vary over time^52^, we also analysed BCR trajectories using a two-state HMM to detect state transitions. In resting B cells where F-actin networks were less dynamic, BCRs exhibited more frequent switching from fast- to slow-moving states. In TLR-activated cells where the F-actin network was more dynamic, switching to the fast state predominated, consistent with BCR mobility being inversely correlated with local F-actin density^2^. Taken together, this suggests that TLR signalling increases the probability of a BCR being in a membrane region where it can diffuse freely, as opposed to being constrained by diffusion barriers that are dependent on the actin cytoskeleton.

Actin-dependent barriers that create confinement zones and limit membrane protein diffusion may take several forms^30^. Submembrane actin filaments may interact with the cytoplasmic domains of transmembrane proteins and impede their diffusion. In addition, transmembrane proteins that are immobilized by tethering to actin filaments can limit receptor diffusion by acting as ‘pickets’^53^. The actin cytoskeleton also maintains the integrity of ‘lipid rafts’ and ‘protein islands’ in which proteins may have reduced diffusion. Increased F-actin severing, as in LPS-activated B cells, would decrease these barriers to receptor diffusion. Cofilin is the major actin-severing protein in LPS-activated B cells^22^ and may therefore be the primary mediator of TLR effects on BCR mobility. Because the Rap1 GTPase regulates cofilin activation^22^, our finding that TLR ligands induce robust Rap1 activation provides a mechanism by which TLR signalling can increase cofilin-mediated F-actin severing.

The enhanced cytoskeletal dynamics caused by TLR signalling may be critical for B-cell responses to APC-bound antigens that are present at low densities. When B cells attach to antigen-bearing APCs, greater BCR mobility would increase the probability of BCRs encountering antigen. At the same time, increased actin turnover would remove diffusion barriers and favour the coalescence of BCRs, or BCR nanoclusters^18^, into microclusters that interact with CD19 to initiate signalling. Further antigen-induced actin turnover^22^,^54^ may increase the frequency of BCR–BCR collisions such that BCR signalling now exceeds the threshold for B-cell activation^46^,^55^. Because of their higher basal levels of BCR mobility and tonic signalling, TLR-activated B cells may require less antigen than resting B cell to reach the ‘tipping point’ at which the activation is triggered^55^.

Changes in actin dynamics and antigen receptor mobility could be a general mechanism for modulating the threshold for lymphocyte activation. Anergic B cells may have a dense, stable actin cytoskeleton that opposes B-cell activation by limiting BCR mobility. In contrast, memory B cells may be primed for activation by having increased actin dynamics, similar to TLR-primed B cells. Memory T cells have preformed TCR oligomers on their surface, which correlates with a lower threshold for their activation^56^. It is not known whether the same is true for memory B cells or whether such antigen-independent receptor clustering requires removal of actin-based barriers that limit antigen receptor aggregation.

Regulators of actin dynamics that limit BCR mobility and antigen-independent BCR signalling may prevent spontaneous B-cell activation that can result in disease. Indeed, patients with loss-of-function mutations in the actin nucleation-promoting factor WASp frequently develop autoantibodies^57^, as do mice lacking WASp in B cells^58^. Dysregulated TLR signalling, which would increase actin dynamics, could have similar effects. Activating mutations in MyD88 that cause ligand-independent TLR signalling occur frequently in diffuse large B-cell lymphomas and are associated with high levels of chronic BCR signalling^59^,^60^. Conversely, the deletion of TLRs or MyD88 reduces autoantibody production in mouse models of autoimmune disease^61^. Thus, cytoskeletal regulators could be therapeutic targets for treating certain types of autoimmune diseases or B-cell malignancies.

Receptor crosstalk via the cytoskeleton may impact many receptors that are constrained by the actin cytoskeleton, for example CD36 (ref. 34) and FcRεI^62^. It may also be critical for integrating signals from receptors that collaborate (for example, the TCR and co-stimulatory receptors) or from opposing activating and inhibitory receptors (for example, the BCR and FcγRIIb, NKG2D and killer cell Ig-like receptors (KIR) on NK cells). FcγRIIb and KIR block the formation of BCR and NKG2D microclusters^63^,^64^, respectively. The inhibitory effect of KIRs on NKG2D microcluster formation is associated with alterations to the actin cytoskeleton and may reflect the inhibition of Vav1 (ref. 65), a key regulator of actin dynamics. Our studies further support a paradigm for receptor crosstalk in which the regulation of cytoskeletal dynamics and receptor mobility modulates receptor signalling and controls the threshold for cellular responses.

## Methods

### B-cell isolation and culture

Splenic B cells were obtained from 6- to 10-week old C57BL/6 mice, MyD88-knockout mice (Jackson Laboratory) or CD-1 mice of either sex using a B-cell isolation kit (Stemcell Technologies) to deplete non-B cells. Experiments performed with B cells from C57BL/6 and CD-1 mice yielded similar results. B cells were cultured in RPMI-1640 supplemented with 10% fetal calf serum, 2 mM glutamine, 1 mM pyruvate, 50 μM 2-mercaptoethanol, 50 U ml^−1^ penicillin and 50 μg ml^−1^ streptomycin (complete medium) along with recombinant murine BAFF (R&D Systems catalogue #2106-BF-010), recombinant murine IL-4 (R&D Systems catalogue #404-ML-010), CpG DNA (ODN1826; InvivoGen catalogue #tlrl-1826) or LPS, as indicated. Similar results were obtained using *E. coli* 0111:B4 LPS (Sigma-Aldrich catalogue #L2630) and *Salmonella enterica* LPS (Sigma-Aldrich catalogue #L9764). Highly enriched populations of CD21^int^CD23^hi^ FO B cells and CD21^hi^CD23^l^° MZ B cells were obtained by fluorescence-activated cell sorting or by using a mouse MZ and FO B-cell isolation kit (Miltenyi Biotec). Animal protocols were approved by the University of British Columbia or Hospital for Sick Children Animal Care Committee.

### Transfection of A20 cells

A20 B-lymphoma cells were from ATCC. Plasmids encoding constitutively active (CA) RhoA (RhoA Q63L) and LifeAct-mCherry were obtained from Addgene (Cambridge, MA). Plasmids encoding WT or catalytically inactive SSH^45^ fused to yellow fluorescent protein were a gift from Dr K. Mizuno (Tohoku University, Sendai, Japan). For transient transfection by electroporation, plasmid DNA was added to 7 × 10^6^ A20 cells before delivering two 20-ms 1350 V pulses with a Neon Transfection System (Life Technologies). Cells were cultured for 24 h before performing SPT.

### APC-induced BCR microcluster formation and BCR signalling

Experiments were performed as described in Freeman *et al*.^22^. Lipofectamine 2000 (Invitrogen) was used to transiently transfect B16F1 melanoma cells (ATCC) with a plasmid encoding the surrogate antigen, which is a fusion protein consisting of a single-chain Fv generated from the 187.1 rat anti-Igκ monoclonal antibody, the hinge and membrane-proximal domains of rat IgG1, and the transmembrane and cytoplasmic domains of the H-2K^b^ MHCI protein^66^. After 24 h, the cells were detached using cell dissociation buffer (0.5 mM EDTA, 100 mM, NaCl, 1 mM glucose, pH 7.4), plated on chamber slides that had been coated with 10 μg ml^−1^ fibronectin, and then cultured for 2 h. B cells were allowed to attach to these APCs for 3 min at 37 °C. B cell:APC conjugates were fixed with 4% paraformaldehyde and the cells were then permeabilized with 0.5% Triton-X-100 in PBS for 3 min and blocked with 2% bovine serum albumin (BSA) in PBS for 20 min. The cells were stained with rabbit anti-pTyr (BD Biosciences catalogue #61009, 1:100 dilution) or rabbit anti-pERK (Cell Signaling Technology catalogue #9101, 1:100 dilution), followed by Alexa568-goat anti-rabbit IgG (Molecular Probes-Invitrogen catalogue #A-11011, 1:200 dilution). Alexa488-goat anti-rat IgG (Molecular Probes-Invitrogen catalogue #A-11006, 1:400 dilution) was used to visualize the single-chain anti-Igκ antibody (that is, the surrogate antigen). Cells that were stained with anti-pERK were also stained with 4′,6-diamidino-2-phenylindole (DAPI) to detect the presence of pERK in the nucleus. Confocal imaging and the quantification of fluorescence signals were performed as described^22^. Images were captured using an Olympus FV1000 confocal microscope with a × 100 objective or with a spinning-disk confocal microscopy system (Quorum Technologies) based on a Zeiss Axiovert 200 M microscope with a × 100 NA 1.45 oil objective, a × 1.5-magnifying lens and a Hamamatsu Photonics C9100-13 camera for image acquisition. ImagePro 6.2 (Media Cybernetics) or Volocity 4.1.1 (PerkinElmer) software was used to analyse confocal images and to quantify fluorescence.

### Cell stimulation and immunoblotting

B cells (5 × 10^6^) were resuspended in 0.4 ml modified HEPES-buffered saline (25 mM sodium HEPES, pH 7.2, 125 mM NaCl, 5 mM KCl, 1 mM CaCl_2_, 1 mM Na_2_HPO_4_, 0.5 mM MgSO_4_, 1 mg ml^−1^ glucose, 2 mM glutamine, 1 mM sodium pyruvate and 50 μM 2-mercaptoethanol). Cells were stimulated in suspension with anti-Igκ light-chain antibodies (SouthernBiotech catalogue #1050-01) or latrunculin (Enzo Life Sciences). Alternatively, the cells were added to wells that had been coated with either the M5/114 anti-MHCII monoclonal antibody (eBioscience catalogue #12-5321) or with anti-Igκ and then blocked with BSA, as described previously^67^. Cells were lysed in RIPA buffer (30 mM Tris-HCl, pH 7.4, 150 mM NaCl, 1% Igepal (Sigma-Aldrich), 0.5% sodium deoxycholate, 0.1% SDS, 2 mM EDTA, 1 mM PMSF (phenylmethylsulphonyl fluoride), 10 μg ml^−1^ leupeptin, 1 μg ml^−1^ aprotinin, 25 mM β-glycerophosphate, 1 μg ml^−1^ microcystin-LR, 1 mM Na_3_VO_4_,). Immunoblotting using ECL detection (GE Life Sciences) was performed as described^22^. Antibodies against cofilin (catalogue #3318), phospho-cofilin (pS3 cofilin; catalogue #3313), ERK (catalogue #9102), phospho-ERK (pERK; catalogue #9101), Akt (catalogue #9272) and phospho-Akt (pAkt; catalogue #9271) were all obtained from Cell Signaling Technologies and used at 1:1,000. For immunoblot images that were cropped for presentation, uncropped images of the entire blot are presented in Supplementary Fig. 14.

### F-actin staining and confocal microscopy

After being fixed and permeabilized as described above, cells were stained with rhodamine–phalloidin (Molecular Probes-Invitrogen) to visualize F-actin. Images were captured using an Olympus FV1000 confocal microscope. FluoView v1.6 and ImagePro software were used to analyse confocal images, generate *z* projections and quantify fluorescence.

### SPT and merge-split analysis

To label single-membrane IgM (mIgM) molecules on primary B cells, or single-membrane IgG (mIgG) molecules on A20 B cells, cells were resuspended to 10^7^ cells ml^−1^, blocked with 5% rat serum for 10 min and then incubated in HBSS (Hanks Balanced Salt Solution) for 5 min with 1 ng ml^−1^ Cy3- or biotin-labelled anti-IgM Fab fragments (for primary B cells), or with 1 ng ml^−1^ biotin-labelled anti-IgG Fab fragments (for A20 cells). All Fab fragments were obtained from Jackson ImmunoResearch Laboratories (catalogue #115-167-020 for Cy3-labelled goat anti-mouse IgM Fab, catalogue #115-067-020 for biotinylated goat anti-mouse IgM Fab, catalogue #115-067-003 for biotinylated goat anti-mouse IgG Fab). When biotinylated Fab fragments were used, the cells were subsequently incubated with streptavidin-655 nm-Qdots (Invitrogen; 1:1,000 dilution) for 5 min and then washed twice with HEPES-buffered RPMI-1640 as a source of free biotin to prevent streptavidin-mediated clustering of the BCR. For merge-split analysis, Cy3-labelled anti-IgM Fab fragments were used at 5 ng ml^−1^. All labelling steps were carried out at 4 °C to minimize lateral mobility and clustering of the BCR. After labelling, the cells were washed and warmed to 37 °C before imaging.

B cells were immobilized for imaging by allowing them to attach for 5 min to coverslips that had been coated with 2 μg cm^−2^ of the M5/114 anti-MHCII antibody (eBioscience) or the indicated densities of anti-Igκ. Live-cell imaging of Qdot-labelled IgM was performed using a Zeiss Axiovert 200 M epifluorescence microscope equipped with a × 100 oil-immersion objective (NA 1.45), a custom-made × 2.4 lens, a 32012 excitation/40 nm-emission filter cube specific for 655 nm-emitting Q dots (Chroma Technology, Bellows Falls, VT) and an Exfo X-Cite 120 light source. A Hamamatsu 9100-13 deep-cooled EM-CCD camera was used for recording and Volocity software was used for image acquisition. Images were acquired continuously at 33 frames s^−1^ for 5–20 s. Imaging of Cy3-labelled mIgM was performed using an Olympus IX81 TIRF microscope equipped with a × 150 oil-immersion objective (NA 1.45) and a Hamamatsu C9100-13 camera. Images were acquired at 10 frames s^−1^ for 5 s. Comparable results were obtained using Cy3 labelling and Qdots.

To validate that the labelling techniques used were detecting single molecules, the fluorescence intensity of regions on the cell surface was measured over time and compared with the fluorescence of soluble monodispersed Cy3-labelled anti-IgM Fab fragments that were imaged using the same microscope settings. The quantized decreases in cell fluorescence due to photobleaching were of identical magnitude for the cell-bound and soluble Cy3-labelled anti-IgM Fab fragments (Supplementary Fig. 3a). Supplementary Fig. 3b shows that that the cell-associated fluorescence signals corresponded to a single Cy3-labelled Fab fragments for ~95% of the events that were detected when the cells were labelled with 1 ng ml^−1^ anti-IgM Fab fragments and for ~75% of the events that were detected when the cells were labelled with 5 ng ml^−1^ anti-IgM Fab fragments. To confirm that only cell surface mIgM was being imaged, the cells were washed in low pH stripping buffer (200 mM acetic acid, 150 mM NaCl, pH 2.8) for 3 min at 37 °C, as described Jaqaman *et al*.^34^, before being transferred to complete medium with 10 mM HEPES. Only cell surface-associated Qdots exposed to the medium should be removed by the low pH treatment. Nearly all of the Qdot fluorescence was removed by this treatment (Supplementary Fig. 3c).

SPT was performed as described previously^26^. In brief, particle positions were determined by fitting Gaussian kernels to local maxima of fluorescence intensities that were detected by imaging. The estimated error in positional accuracy was determined for random particles using an algorithm that takes into account the shape of the Gaussian curve that was fit to the particle image (see Supplementary Fig. 4). Particles were tracked using a two-step algorithm to generate complete trajectories by closing gaps and capturing merging and splitting events. First, the algorithm was used to link particle positions between consecutive frames using optimized spatial assignments that were applied identically for all videos generated using either Qdot- or Cy3-labelled particles. Second, the algorithm generated complete particle trajectories by linking track segments from end-to-start in order to close gaps resulting from temporary particle disappearance due to Qdot “blinking”. The cost functions employed to weigh competing particle and track segment assignments were based on distance and intensity, and on motion models that aided tracking by using the Kalman filter algorithm to allow particle position propagation. Tracks lasting at least five frames were retained for trajectory analysis.

### Single-state analysis of BCR trajectories

The single-state model assumes that displacements arise from a two-dimensional Brownian (free) diffusion process. Diffusion coefficients (*D*) were calculated for individual tracks using a maximum likelihood estimation (mle) approach described by Das *et al*.^29^. A single diffusion coefficient (mle) for the entire trajectory was derived using the expression 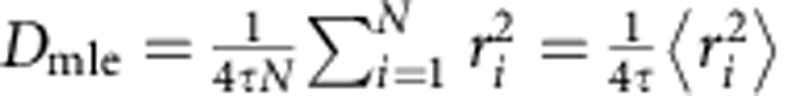, in which *τ* is the time interval between successive observations of the particle position (that is, the inverse of the frame rate), *N* is the number of time intervals and 
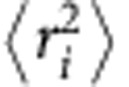
 is the mean squared step size within each track.

### Two-state HMM analysis of BCR trajectories

We also analysed BCR tracks using a two-state HMM^29^, which assumes that individual trajectories consist of slow and fast segments. It uses a likelihood maximization scheme to compute the distribution of diffusion coefficients for each state, as well as the probability of transitions between the two states that best match the observed trajectories. Per-frame transition probabilities were converted to first-order transition rates by multiplying them by the frame rate.

### MSS analysis of BCR trajectories

MSS analysis of particle displacements was used to classify BCR trajectories as reflecting free diffusion, confined or subdiffusive behaviour, or directed motion^26^,^32^,^33^. In the single-parameter description of motion that can be used to characterize free (Brownian) diffusion, the individual steps that comprise the motion are assumed to form a normal distribution. In this case, the higher moments (powers) of the step-length distribution increase (scale) linearly with the square root of the order of the moment. The MSS analysis improves the ability to detect deviations from this behaviour. Particles that are confined or that exhibit subdiffusive behaviour are characterized by step-length moments that grow more slowly with the order of the moment than for free diffusion. Particles exhibiting directed motion exhibit the opposite scaling behaviour. By considering higher-order moments, the MSS analysis allows more accurate classification of trajectories as either free or confined than considering only the second-order moment by assessing the change in MSD over time. The MSS analysis was applied to full two-dimensional displacements and to one-dimensional displacements in the case of linear trajectories. Particle diffusion coefficients were calculated from the MSS analysis. For each trajectory, the squared displacement is calculated for every 1-, 2-, 3-, 4- and 5-frame interval (with frames being 30 ms apart). For example, the set of three-step intervals includes the values for frames 1 to 4, 2 to 5, 3 to 6, …. and (*n*−3) to *n*, where *n* is the number of frames in the trajectory. The average MSD for each time-step interval was determined and fitted to a straight line of MSD versus the time interval to calculate the diffusion coefficient (*D*) according to the relationship MSD=4*D*Δ*t* (or MSD=4*D*Δ*t*^*α*^ for subdiffusive/confined particles, in which case *α*<1). A correction term (*K*) defined by Qian *et al*.^68^ corrects for the fact that the values determined for different frame intervals are not all statistically independent because of the overlap between the segments. The confinement dimension for confined and linear trajectories was derived via eigenvalue decomposition of the variance–covariance matrix of particle positions along each trajectory as done by Jaqaman *et al*.^34^ In brief, the confinement area was determined using an algorithm that fits all the positions of a given track into the minimum size circle and then determines the radius of this circle.

### Scanning electron microscopy

B cell:APC conjugates, or B cells that were immobilized on anti-MHCII-coated coverslips, were fixed with glutaraldehyde and processed for scanning electron microscopy exactly as described previously^67^. Images were obtained using the Hitachi S-4700 field emission scanning electron microscope in the University of British Columbia BioImaging Facility.

### Determination of G-actin/F-actin ratio

Cells were lysed and F-actin filaments were stabilized using a G-actin/F-actin *in vivo* assay kit (Cytoskeleton Inc.), according the manufacturer’s instructions. The cell extracts were separated into soluble and insoluble fractions by ultracentrifugation (100,000 *g*, 1 h, 37 °C). These fractions were analysed by immunoblotting with a β-actin antibody (Sigma-Aldrich catalogue #A2228, 1:3,000 dilution) to detect soluble G-actin and insoluble F-actin, as described previously^22^.

### *In vitro* actin polymerization activity in cell extracts

As described previously^22^, cells were lysed in RIPA buffer containing 1 mM ATP and then sonicated for 1 min to destroy pre-existing F-actin. The sonicated cell extracts were then incubated at 37 °C for 10 min to allow *in vitro* actin polymerization. After adding F-actin stabilization buffer (Cytoskeleton Inc.) for 10 min at 37 °C, the cell extracts were separated into soluble and insoluble fractions as described above. The amount of soluble G-actin converted into insoluble F-actin during the 10- min *in vitro* incubation period was assessed by immunoblotting with a β-actin antibody.

### *In vitro* F-actin severing activity

As described by Freeman *et al*.^22^, chamber slides (ibidi) were incubated with 20 μg ml^−1^ anti-biotin antibodies in ISAP buffer (20 mM Tris-HCl, pH 7.5, 5 mM EGTA, 2 mM MgCl_2_, 50 mM KCl, 1 mM ATP, 1 mM DTT (dithiothreitol)) for 1 h at room temperature and then blocked with 0.5 mg ml^−1^ BSA for 5 min. Biotinylated fluorescent F-actin filaments were generated by incubating 0.2 μM Alexa488-actin, 0.2 μM biotinylated actin and 0.4 μM unlabelled actin (all from Cytoskeleton Inc.) in ISAP buffer for 1 h at 20 °C. The resulting actin filaments were added to the anti-biotin-coated chamber slide for 5 min. After washing with ISAP buffer containing 5 mg ml^−1^ BSA, chambers were imaged by confocal microscopy to determine the pre-assay fluorescence intensity of adhered F-actin filaments. Cells were lysed in 0.5 ml lysis buffer (20 mM Tris-HCl, pH 8, 5 mM EDTA, 0.5 mM MgCl_2_, 0.5% Triton-X-100, 0.5 mM ATP, 5 mg ml^−1^ BSA, 6 mg ml^−1^ glucose, 100 mM DTT, 1 mM PMSF, 10 μg ml^−1^ leupeptin, 1 μg ml^−1^ aprotinin, 1 μg ml^−1^ pepstatin, 1 mM Na_3_VO_4_, 25 mM β-glycerophosphate) for 20 min on ice. Insoluble material was removed by centrifugation, after which the cell extracts were added to the chamber slides for 5 min. The chambers were then washed and imaged to determine the fluorescence intensity of the remaining F-actin filaments. Fluorescence intensities were quantified using ImagePro software (Media Cybernetics).

### Actin polymerization in permeabilized cells

Cells were rendered semipermeable by a 10-s incubation with permeabilization buffer (20 mM HEPES, pH 7.5, 138 mM KCl, 4 mM MgCl_2_, 3 mM EGTA, 0.4 mg ml^−1^ saponin, 1% BSA, 1 mM ATP) in the presence of 0.4 μM Alexa488-actin (Cytoskeleton Inc.), as described by Freeman *et al*.^22^. The cells were then fixed with 4% paraformaldehyde in PBS for 20 min at 20 °C, stained with rhodamine–phalloidin for 20 min and imaged by confocal microscopy. Fluorescence intensities were quantified using ImagePro software and the ratio of Alexa 488 actin/F-actin fluorescence was calculated for individual cells.

### Measurement of active Rac1 and RhoA

The amount of active GTP-bound Rac1 or RhoA in B-cell extracts was determined using G-LISA kits (Cytoskeleton Inc.) according to the manufacturer’s instructions.

### Cofilin peptides and RhoA-activating peptides

Cell-permeable peptides that inhibit F-actin severing by cofilin^43^ were synthesized by Biopeptide Inc. (San Diego, CA). The M (CDYKDDDDKMASGVAVSDGVIK) and W (CDYKDDDDKWAPESAPLKSKM) peptides that correspond to actin-binding regions from cofilin, as well as the Q peptide (CDYKDDDDKWAPESAPLQSQM) in which key lysine residues in the W peptide were changed to glutamates so as to ablate F-actin binding^43^, were coupled to penetratin (KKWKMRRNQFWIKIQR-3-nitro-2-pyridinesulfenyl). Penetratin-peptide conjugates were purified by HPLC, and peptide masses were confirmed by mass spectrometry. B cells were resuspended in cold PBS and incubated with 5 μM each of the M and W peptides or with 5 μM of the Q peptide for 10 min on ice before placing the cells at 37 °C for 5 min and then adhering them to anti-MHCII-coated coverslips or adding them to APCs. The cell-permeable CN03 RhoA activator II (ref. 44) (Cytoskeleton Inc.), which activates RhoA in intact cells^69^, was obtained from Cytoskeleton Inc. B cells were incubated with 1 μg ml^−1^ of the CN03 RhoA activator in complete medium for 2–3 h at 37 °C, then resuspended in HBSS, warmed to 37 °C and allowed to attach to anti-MHCII-coated coverslips or APCs.

### Statistical analysis

Student’s two-tailed unpaired *t*-test was used to compare sets of matched samples.

## Author contributions

S.A.F. designed and performed the experiments, analysed the data and wrote the manuscript; V.J. designed experiments and performed the MSS analysis on SPT data; K.C. designed, performed and analysed key experiments; B.E.H, L.A. and M.L.G. performed key experiments; H.S.W. performed MSS analysis on SPT data; R.D. and D.C. developed and applied analytical tools for the single-state and two-state analyses of SPT data; C.D.R. assisted with data analysis and with writing the manuscript; S.G. and M.R.G. designed the experiments, analysed data and wrote the manuscript.

## Additional information

**How to cite this article:** Freeman, S. A. *et al*. Toll-like receptor ligands sensitize B-cell receptor signalling by reducing actin-dependent spatial confinement of the receptor. *Nat. Commun.* 6:6168 doi: 10.1038/ncomms7168 (2015).

## Supplementary Material

Supplementary Information Supplementary Figures 1-16, Supplementary Tables 1-2 and Supplementary References

Supplementary Movie 1 mIgM-containing BCRs on BAFF-cultured B cells exhibit mostly confined motion

Supplementary Movie 2 LPS decreases the confinement of mIgM-containing BCRs

Supplementary Movie 3 CpG DNA decreases the confinement of mIgM-containing BCRs

Supplementary Movie 4 The actin cytoskeleton confines BCR mobility in resting B cells

Supplementary Movie 5 The actin cytoskeleton contributes to BCR confinement in LPS-treated B cells

Supplementary Movie 6 Blocking cofilin-mediated actin severing inhibits BCR mobility

Supplementary Movie 7 Increasing RhoA activity decreases BCR mobility

## References

[b1] Jaqaman K. & Grinstein S. Regulation from within: the cytoskeleton in transmembrane signaling. Trends Cell Biol. 22, 515–526 (2012). 22917551 10.1016/j.tcb.2012.07.006 PMC3754899

[b2] Treanor B. . The membrane skeleton controls diffusion dynamics and signaling through the B cell receptor. Immunity 32, 187–199 (2010). 20171124 10.1016/j.immuni.2009.12.005 PMC2984614

[b3] Song W., Liu C. & Upadhyaya A. The pivotal position of the actin cytoskeleton in the initiation and regulation of B cell receptor activation. Biochim. Biophys. Acta 1838, 569–578 (2013). 23886914 10.1016/j.bbamem.2013.07.016 PMC3877175

[b4] DeFranco A. L., Rookhuizen D. C. & Hou B. Contribution of Toll-like receptor signaling to germinal center antibody responses. Immunol. Rev. 247, 64–72 (2012). 22500832 10.1111/j.1600-065X.2012.01115.x PMC3334874

[b5] Kawai T. & Akira S. Toll-like receptors and their crosstalk with other innate receptors in infection and immunity. Immunity 34, 637–650 (2011). 21616434 10.1016/j.immuni.2011.05.006

[b6] Hou B. . Selective utilization of Toll-like receptor and MyD88 signaling in B cells for enhancement of the antiviral germinal center response. Immunity 34, 375–384 (2011). 21353603 10.1016/j.immuni.2011.01.011 PMC3064721

[b7] Eckl-Dorna J. & Batista F. D. BCR-mediated uptake of antigen linked to TLR9 ligand stimulates B-cell proliferation and antigen-specific plasma cell formation. Blood 113, 3969–3977 (2009). 19144984 10.1182/blood-2008-10-185421

[b8] Batista F. D. & Harwood N. E. The who, how and where of antigen presentation to B cells. Nat. Rev. Immunol. 9, 15–27 (2009). 19079135 10.1038/nri2454

[b9] Cyster J. G. B cell follicles and antigen encounters of the third kind. Nat. Immunol. 11, 989–996 (2010). 20959804 10.1038/ni.1946

[b10] Heesters B. A. . Endocytosis and recycling of immune complexes by follicular dendritic cells enhances B cell antigen binding and activation. Immunity 38, 1164–1175 (2013). 23770227 10.1016/j.immuni.2013.02.023 PMC3773956

[b11] Fleire S. J. . B cell ligand discrimination through a spreading and contraction response. Science 312, 738–741 (2006). 16675699 10.1126/science.1123940

[b12] Weber M. . Phospholipase C-γ2 and Vav cooperate within signaling microclusters to propagate B cell spreading in response to membrane-bound antigen. J. Exp. Med. 205, 853–868 (2008). 18362175 10.1084/jem.20072619 PMC2292224

[b13] Depoil D. . CD19 is essential for B cell activation by promoting B cell receptor-antigen microcluster formation in response to membrane-bound ligand. Nat. Immunol. 9, 63–72 (2008). 18059271 10.1038/ni1547

[b14] Tolar P., Sohn H. W., Liu W. & Pierce S. K. The molecular assembly and organization of signaling active B-cell receptor oligomers. Immunol. Rev. 232, 34–41 (2009). 19909354 10.1111/j.1600-065X.2009.00833.x

[b15] Treanor B., Harwood N. E. & Batista F. D. Microsignalosomes: spatially resolved receptor signalling. Biochem. Soc. Trans. 37, 1014–1018 (2009). 19754442 10.1042/BST0371014

[b16] Carrasco Y. R., Fleire S. J., Cameron T., Dustin M. L. & Batista F. D. LFA-1/ICAM-1 interaction lowers the threshold of B cell activation by facilitating B cell adhesion and synapse formation. Immunity 20, 589–599 (2004). 15142527 10.1016/s1074-7613(04)00105-0

[b17] Carrasco Y. R. & Batista F. D. B-cell activation by membrane-bound antigens is facilitated by the interaction of VLA-4 with VCAM-1. EMBO J. 25, 889–899 (2006). 16456548 10.1038/sj.emboj.7600944 PMC1383545

[b18] Mattila P. K. . The actin and tetraspanin networks organize receptor nanoclusters to regulate B cell receptor-mediated signaling. Immunity 38, 461–474 (2013). 23499492 10.1016/j.immuni.2012.11.019

[b19] Harwood N. E. & Batista F. D. The cytoskeleton coordinates the early events of B-cell activation. Cold Spring Harb. Perspect. Biol 3,, pii: a002360 (2011). 10.1101/cshperspect.a002360 PMC3039531 21047917

[b20] Treanor B., Depoil D., Bruckbauer A. & Batista F. D. Dynamic cortical actin remodeling by ERM proteins controls BCR microcluster organization and integrity. J. Exp. Med. 208, 1055–1068 (2011). 21482698 10.1084/jem.20101125 PMC3092358

[b21] Lam K. P., Kuhn R. & Rajewsky K. *In vivo* ablation of surface immunoglobulin on mature B cells by inducible gene targeting results in rapid cell death. Cell 90, 1073–1083 (1997). 9323135 10.1016/s0092-8674(00)80373-6

[b22] Freeman S. A. . Cofilin-mediated F-actin severing is regulated by the Rap GTPase and controls the cytoskeletal dynamics that drive lymphocyte spreading and BCR microcluster formation. J. Immunol. 187, 5887–5900 (2011). 22068232 10.4049/jimmunol.1102233

[b23] Liu C. . Actin reorganization is required for the formation of polarized B cell receptor signalosomes in response to both soluble and membrane-associated antigens. J. Immunol. 188, 3237–3246 (2012). 22387556 10.4049/jimmunol.1103065 PMC3312033

[b24] Ketchum C., Miller H., Song W. & Upadhyaya A. Ligand mobility regulates B cell receptor clustering and signaling activation. Biophys. J. 106, 26–36 (2014). 24411234 10.1016/j.bpj.2013.10.043 PMC3907214

[b25] Tolar P., Hanna J., Krueger P. D. & Pierce S. K. The constant region of the membrane immunoglobulin mediates B cell-receptor clustering and signaling in response to membrane antigens. Immunity 30, 44–55 (2009). 19135393 10.1016/j.immuni.2008.11.007 PMC2656684

[b26] Jaqaman K. . Robust single-particle tracking in live-cell time-lapse sequences. Nat. Methods 5, 695–702 (2008). 18641657 10.1038/nmeth.1237 PMC2747604

[b27] Liu W., Meckel T., Tolar P., Sohn H. W. & Pierce S. K. Intrinsic properties of immunoglobulin IgG1 isotype-switched B cell receptors promote microclustering and the initiation of signaling. Immunity 32, 778–789 (2010). 20620943 10.1016/j.immuni.2010.06.006 PMC2904325

[b28] Liu W., Meckel T., Tolar P., Sohn H. W. & Pierce S. K. Antigen affinity discrimination is an intrinsic function of the B cell receptor. J. Exp. Med. 207, 1095–1111 (2010). 20404102 10.1084/jem.20092123 PMC2867278

[b29] Das R., Cairo C. W. & Coombs D. A hidden Markov model for single particle tracks quantifies dynamic interactions between LFA-1 and the actin cytoskeleton. PLoS Comput. Biol. 5, e1000556 (2009). 19893741 10.1371/journal.pcbi.1000556 PMC2768823

[b30] Kusumi A., Suzuki K. G., Kasai R. S., Ritchie K. & Fujiwara T. K. Hierarchical mesoscale domain organization of the plasma membrane. Trends Biochem. Sci. 36, 604–615 (2011). 21917465 10.1016/j.tibs.2011.08.001

[b31] Kusumi A., Sako Y. & Yamamoto M. Confined lateral diffusion of membrane receptors as studied by single particle tracking (nanovid microscopy). Effects of calcium-induced differentiation in cultured epithelial cells. Biophys. J. 65, 2021–2040 (1993). 8298032 10.1016/S0006-3495(93)81253-0 PMC1225938

[b32] Ferrari R., Manfoi A. J. & Young W. R. Strongly and weakly self-similar diffusion. Physica D 154, 111–137 (2001).

[b33] Ewers H. . Single-particle tracking of murine polyoma virus-like particles on live cells and artificial membranes. Proc. Natl Acad. Sci. USA 102, 15110–15115 (2005). 16219700 10.1073/pnas.0504407102 PMC1257700

[b34] Jaqaman K. . Cytoskeletal control of CD36 diffusion promotes its receptor and signaling function. Cell 146, 593–606 (2011). 21854984 10.1016/j.cell.2011.06.049 PMC3160624

[b35] Wong H. S. . Cytoskeletal confinement of CX3CL1 limits its susceptibility to proteolytic cleavage by ADAM10. Mol. Biol. Cell 25, 3884–3899 (2014). 25253723 10.1091/mbc.E13-11-0633 PMC4244198

[b36] Jaumouille V. . Actin cytoskeleton reorganization by Syk regulates Fcγ receptor responsiveness by increasing its lateral mobility and clustering. Dev. Cell 29, 534–546 (2014). 24914558 10.1016/j.devcel.2014.04.031 PMC4083245

[b37] Barrio L., Saez de Guinoa J. & Carrasco Y. R. TLR4 signaling shapes B cell dynamics via MyD88-dependent pathways and Rac GTPases. J. Immunol. 191, 3867–3875 (2013). 23997213 10.4049/jimmunol.1301623

[b38] Husson C., Renault L., Didry D., Pantaloni D. & Carlier M. F. Cordon-Bleu uses WH2 domains as multifunctional dynamizers of actin filament assembly. Mol. Cell 43, 464–477 (2011). 21816349 10.1016/j.molcel.2011.07.010

[b39] Ichetovkin I., Grant W. & Condeelis J. Cofilin produces newly polymerized actin filaments that are preferred for dendritic nucleation by the Arp2/3 complex. Curr. Biol. 12, 79–84 (2002). 11790308 10.1016/s0960-9822(01)00629-7

[b40] Van Troys M. . Ins and outs of ADF/cofilin activity and regulation. Eur. J. Cell Biol. 87, 649–667 (2008). 18499298 10.1016/j.ejcb.2008.04.001

[b41] McLeod S. J., Shum A. J., Lee R. L., Takei F. & Gold M. R. The Rap GTPases regulate integrin-mediated adhesion, cell spreading, actin polymerization, and Pyk2 tyrosine phosphorylation in B lymphocytes. J. Biol. Chem. 279, 12009–12019 (2004). 14701796 10.1074/jbc.M313098200

[b42] Dransfield I., Cabanas C., Craig A. & Hogg N. Divalent cation regulation of the function of the leukocyte integrin LFA-1. J. Cell Biol. 116, 219–226 (1992). 1346139 10.1083/jcb.116.1.219 PMC2289255

[b43] Eibert S. M. . Cofilin peptide homologs interfere with immunological synapse formation and T cell activation. Proc. Natl Acad. Sci. USA 101, 1957–1962 (2004). 14762171 10.1073/pnas.0308282100 PMC357034

[b44] Schmidt G. . Gln 63 of Rho is deamidated by *Escherichia coli* cytotoxic necrotizing factor-1. Nature 387, 725–729 (1997). 9192900 10.1038/42735

[b45] Niwa R., Nagata-Ohashi K., Takeichi M., Mizuno K. & Uemura T. Control of actin reorganization by Slingshot, a family of phosphatases that dephosphorylate ADF/cofilin. Cell 108, 233–246 (2002). 11832213 10.1016/s0092-8674(01)00638-9

[b46] Treanor B. & Batista F. D. Organisation and dynamics of antigen receptors: implications for lymphocyte signalling. Curr. Opin. Immunol. 22, 299–307 (2010). 20434893 10.1016/j.coi.2010.03.009

[b47] Khan W. N. . Defective B cell development and function in Btk-deficient mice. Immunity 3, 283–299 (1995). 7552994 10.1016/1074-7613(95)90114-0

[b48] Engel P. . Abnormal B lymphocyte development, activation, and differentiation in mice that lack or overexpress the CD19 signal transduction molecule. Immunity 3, 39–50 (1995). 7542548 10.1016/1074-7613(95)90157-4

[b49] Jumaa H. . Abnormal development and function of B lymphocytes in mice deficient for the signaling adaptor protein SLP-65. Immunity 11, 547–554 (1999). 10591180 10.1016/s1074-7613(00)80130-2

[b50] Hebeis B., Vigorito E., Kovesdi D. & Turner M. Vav proteins are required for B-lymphocyte responses to LPS. Blood 106, 635–640 (2005). 15811961 10.1182/blood-2004-10-3919

[b51] Cerutti A., Cols M. & Puga I. Marginal zone B cells: virtues of innate-like antibody-producing lymphocytes. Nat. Rev. Immunol. 13, 118–132 (2013). 23348416 10.1038/nri3383 PMC3652659

[b52] Kusumi A. . Dynamic organizing principles of the plasma membrane that regulate signal transduction: commemorating the fortieth anniversary of Singer and Nicolson's fluid-mosaic model. Annu. Rev. Cell Dev. Biol. 28, 215–250 (2012). 22905956 10.1146/annurev-cellbio-100809-151736

[b53] Cairo C. W. . Dynamic regulation of CD45 lateral mobility by the spectrin-ankyrin cytoskeleton of T cells. J. Biol. Chem. 285, 11392–11401 (2010). 20164196 10.1074/jbc.M109.075648 PMC2857017

[b54] Hao S. & August A. Actin depolymerization transduces the strength of B-cell receptor stimulation. Mol. Biol. Cell 16, 2275–2284 (2005). 15728723 10.1091/mbc.E04-10-0881 PMC1087234

[b55] Pierce S. K. & Liu W. The tipping points in the initiation of B cell signalling: how small changes make big differences. Nat. Rev. Immunol. 10, 767–777 (2010). 20935671 10.1038/nri2853 PMC3406597

[b56] Kumar R. . Increased sensitivity of antigen-experienced T cells through the enrichment of oligomeric T cell receptor complexes. Immunity 35, 375–387 (2011). 21903423 10.1016/j.immuni.2011.08.010

[b57] Dupuis-Girod S. . Autoimmunity in Wiskott-Aldrich syndrome: risk factors, clinical features, and outcome in a single-center cohort of 55 patients. Pediatrics 111, e622–e627 (2003). 12728121 10.1542/peds.111.5.e622

[b58] Becker-Herman S. . WASp-deficient B cells play a critical, cell-intrinsic role in triggering autoimmunity. J. Exp. Med. 208, 2033–2042 (2011). 21875954 10.1084/jem.20110200 PMC3182055

[b59] Ngo V. N. . Oncogenically active MYD88 mutations in human lymphoma. Nature 470, 115–119 (2011). 21179087 10.1038/nature09671 PMC5024568

[b60] Davis R. E. . Chronic active B-cell-receptor signalling in diffuse large B-cell lymphoma. Nature 463, 88–92 (2010). 20054396 10.1038/nature08638 PMC2845535

[b61] Green N. M. & Marshak-Rothstein A. Toll-like receptor driven B cell activation in the induction of systemic autoimmunity. Semin. Immunol. 23, 106–112 (2011). 21306913 10.1016/j.smim.2011.01.016 PMC3070769

[b62] Andrews N. L. . Actin restricts FcεRI diffusion and facilitates antigen-induced receptor immobilization. Nat. Cell Biol. 10, 955–963 (2008). 18641640 10.1038/ncb1755 PMC3022440

[b63] Liu W., Sohn H. W., Tolar P., Meckel T. & Pierce S. K. Antigen-induced oligomerization of the B cell receptor is an early target of FcγRIIB inhibition. J. Immunol. 184, 1977–1989 (2010). 20083655 10.4049/jimmunol.0902334 PMC2931798

[b64] Abeyweera T. P., Merino E. & Huse M. Inhibitory signaling blocks activating receptor clustering and induces cytoskeletal retraction in natural killer cells. J. Cell Biol. 192, 675–690 (2011). 21339333 10.1083/jcb.201009135 PMC3044118

[b65] Stebbins C. C. . Vav1 dephosphorylation by the tyrosine phosphatase SHP-1 as a mechanism for inhibition of cellular cytotoxicity. Mol. Cell. Biol. 23, 6291–6299 (2003). 12917349 10.1128/MCB.23.17.6291-6299.2003 PMC180957

[b66] Ait-Azzouzene D. . An immunoglobulin Cκ-reactive single chain antibody fusion protein induces tolerance through receptor editing in a normal polyclonal immune system. J. Exp. Med. 201, 817–828 (2005). 15738053 10.1084/jem.20041854 PMC2212821

[b67] Lin K. B. . The Rap GTPases regulate B cell morphology, immune-synapse formation, and signaling by particulate B cell receptor ligands. Immunity 28, 75–87 (2008). 18191594 10.1016/j.immuni.2007.11.019

[b68] Qian H., Sheetz M. P. & Elson E. L. Single particle tracking. Analysis of diffusion and flow in two-dimensional systems. Biophys. J. 60, 910–921 (1991). 1742458 10.1016/S0006-3495(91)82125-7 PMC1260142

[b69] Valtcheva N., Primorac A., Jurisic G., Hollmen M. & Detmar M. The orphan adhesion G protein-coupled receptor GPR97 regulates migration of lymphatic endothelial cells via the small GTPases RhoA and Cdc42. J. Biol. Chem. 288, 35736–35748 (2013). 24178298 10.1074/jbc.M113.512954 PMC3861625

