# Supplementary material for: Toll-like receptor ligands sensitize B-cell receptor signalling by reducing actin-dependent spatial confinement of the receptor
Source: Nat Commun. 2015 Feb 3;6:6168. doi: 10.1038/ncomms7168 (PMC4327415; doi:10.1038/ncomms7168)
Supplement: Supplementary Information — Supplementary Figures 1-16, Supplementary Tables 1-2 and Supplementary References [file ncomms7168-s1.pdf]

## Supplementary Figures and Tables

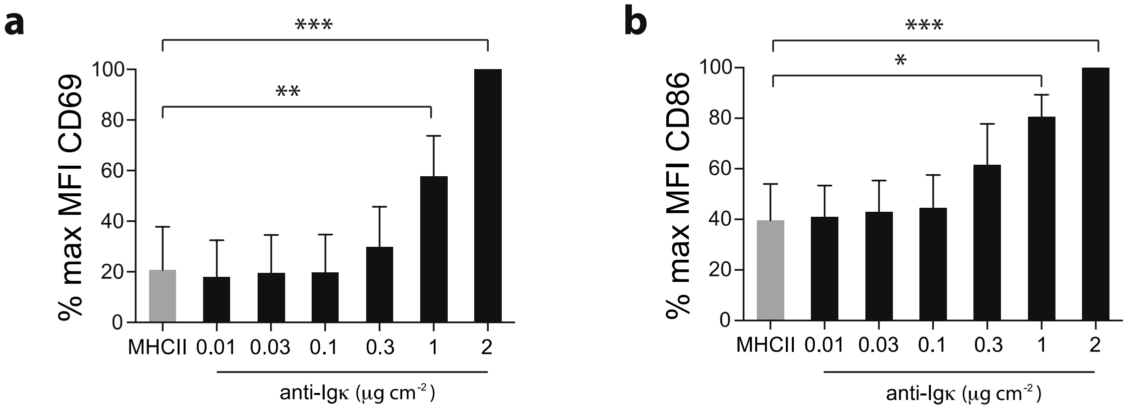


**Supplementary Figure 1 | Dose-response for upregulation of CD69 and CD86 by immobilized anti-Igκ antibodies.** B cells were cultured overnight with 5 ng $ml^{-1}$ BAFF and then added to wells that had been coated with the indicated densities of anti-MHCII (non-stimulatory antibody; see **Supplementary Fig. 2**) or anti-Igκ. After 8 h, the cells were detached from the wells by vigorous pipetting and then stained with either anti-CD69-PE-Cy7 (**a**) or anti-CD86-APC (**b**) before being analyzed by flow cytometry. FlowJo software was used to determine the mean fluorescence intensity (MFI) for each sample. The data are presented as a percent of the MFI for the 2 μg $cm^{-2}$ anti-Igκ sample. Each bar represents the mean $\pm$ s.e.m. for 4-5 independent experiments. ***$P$<0.001; **$P$<0.01; *$P$<0.05, as determined using Student's 2-tailed unpaired t-test. The MFI values for cells plated on anti-Igκ densities less than 1 μg $ml^{-1}$ were not significantly different than those for cells that were plated on the non-stimulatory anti-MHCII antibody.

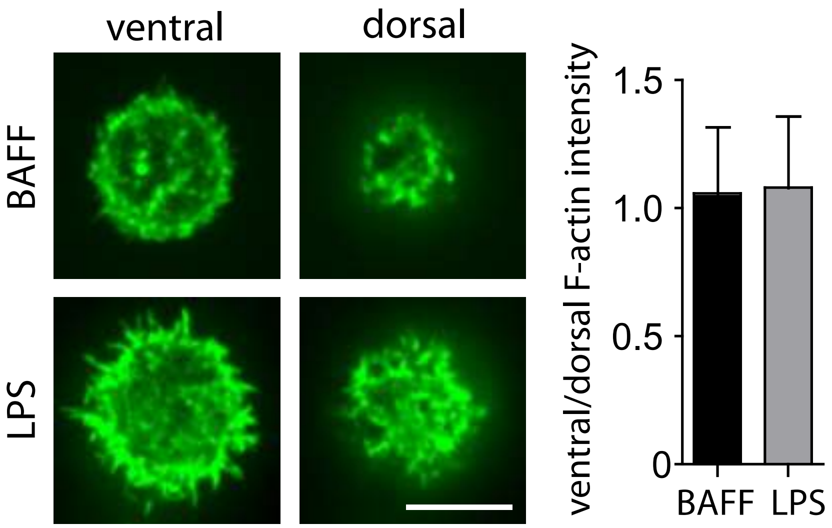


**Supplementary Figure 2 | Immobilized anti-MHCII antibodies do not alter cytoskeletal organization in B cells.** Primary B cells that had been cultured overnight with 5 ng ml$^{-1}$ BAFF or with BAFF + 5 µg ml$^{-1}$ LPS were adhered to anti-MHCII-coated coverslips and stained with Alexa488-phalloidin. F-actin at the ventral and dorsal surfaces of the cell was visualized by confocal microscopy. Scale bar, 5 µm. The ratio of F-actin fluorescence intensity per unit area at the ventral and dorsal surfaces was determined for >10 cells in each experiment and the mean ± s.e.m. for 3 experiments is shown. For both the BAFF- and LPS-cultured B cells, the F-actin densities on the ventral and dorsal sides of the cells remained nearly identical when the cells were plated on anti-MHCII antibodies. This, in combination with the absence of cell spreading and F-actin clearance on the ventral side of the cell that was in contact with the anti-MHCII antibodies, indicates that this is a non-stimulatory condition. This is in contrast to the dramatic spreading and F-actin clearance induced by plating cells on anti-Ig antibodies[1, 2].

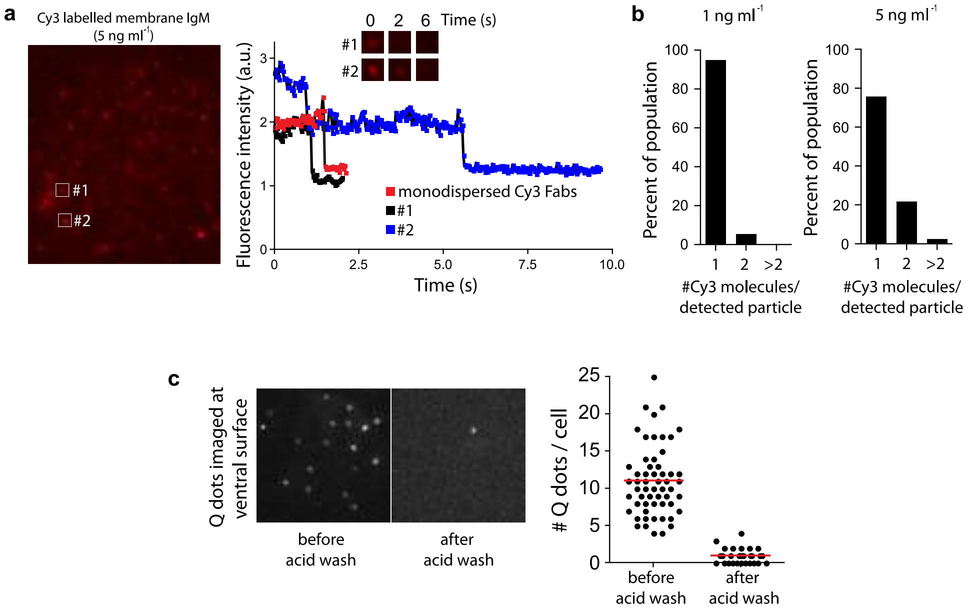


**Supplementary Figure 3 | Single-particle labeling of BCRs on the B cell surface.**

(**a**) B cells were labeled on ice with 5 ng ml$^{-1}$ Cy3-labeled anti-IgM Fab fragments. The cells were adhered to anti-MHCII-coated coverslips and then imaged by total internal reflection microscopy (TIRF) at 33 frames s$^{-1}$ for 10 s with laser settings that allowed photobleaching to be observed. A still image from a representative video is shown (left panel). Fluorescence intensities in the indicated regions are compared to that for monodispersed soluble Cy3-labeled anti-IgM Fabs that were imaged using the same settings (right panel). Single-particle labeling is indicated by a single quantized fluorescence decrease (e.g. region #1, black trace) of similar magnitude to that for monodispersed Cy3-anti-IgM Fabs (red trace). Although the majority of spots contained a single Cy3-labeled Fab, some contained >1, e.g. region #2 (blue trace) contained two.

(**b**) This photobleaching analysis was performed on >300 random particles. When cells were labeled with 5 ng ml$^{-1}$ Cy3-anti-IgM Fabs, ~75% of identified particles contained only one Fab. When cells were labeled with 1 ng ml$^{-1}$ Cy3-anti-IgM Fabs (the concentration used in Qdot SPT experiments), ~95% of particles contained only one Fab. The linear relationship between the Fab concentration and the number of particles containing multiple Cy3 molecules (~5% for 1 ng ml$^{-1}$ Fab; ~25% for 5 ng ml$^{-1}$) is consistent with labeling of mostly single BCRs.

(**c**) B cells were labeled with 1 ng ml$^{-1}$ biotin-anti-IgM Fabs plus streptavidin-655 nm Qdots before imaging the ventral surface. The number of Qdots at the ventral surface was determined before and after washing the cells with a low-pH stripping buffer. Acid-dissociable Qdots are assumed to be on the cell surface.

Note that anti-IgG Fabs did not label primary splenic B cells and that anti-IgM Fabs did not label the mIgG$^{+}$ A20 B-lymphoma cells, demonstrating the specificity of the reagents used for SPT of BCRs. Qdots alone did not bind to B cells. Moreover, when B cells were labeled with Qdots, washed, and then adhered to anti-MHCII-coated coverslips, we did not observe any Qdots that were not associated with B cells.

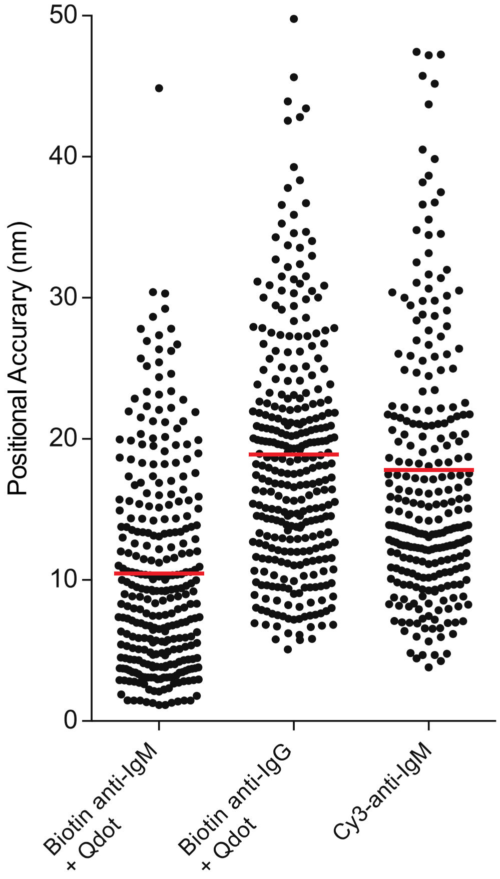


**Supplementary Figure 4 | Estimation of the error in determining particle position (positional accuracy).** *Ex vivo* B cells were labeled with 1 ng ml$^{-1}$ Cy3-labeled anti-IgM Fab fragments or with 1 ng ml$^{-1}$ biotinylated anti-IgM Fab fragments plus streptavidin-655 nm Qdots. A20 B-lymphoma cells were labeled with 1 ng ml$^{-1}$ biotinylated anti-IgG Fab fragments plus streptavidin-655 nm Qdots. The cells were adhered to anti-MHCII-coated coverslips before being imaged in real time. Single-particle tracking (SPT) using u-track software was performed as described[3]. Particle positions were determined by fitting Gaussian kernels to local maxima of fluorescence intensities. The positional accuracy was calculated from the width of the Gaussian curve that was fit to the particle intensity profile[3]. For each labeling condition, this value was determined for >300 random particles. Each point represents the estimated positional accuracy for an individual particle. Bars indicate mean values. The mean positional accuracy for particles visualized by labeling primary B cells with biotinylated anti-IgM Fab fragments plus Qdots was 10.5 nm. This labeling condition was employed for all experiments with the following exceptions. For experiments involving A20 B-lymphoma cells (**Fig. 5i, Fig. 6**) BCRs were labeled with biotinylated anti-IgG Fab fragments plus Qdots (mean positional accuracy of 18.9 nm). For the experiments shown in **Supplementary Fig. 15**, B cells were labeled with Cy3-anti-IgM Fab fragments (mean positional accuracy of 17.8 nm).

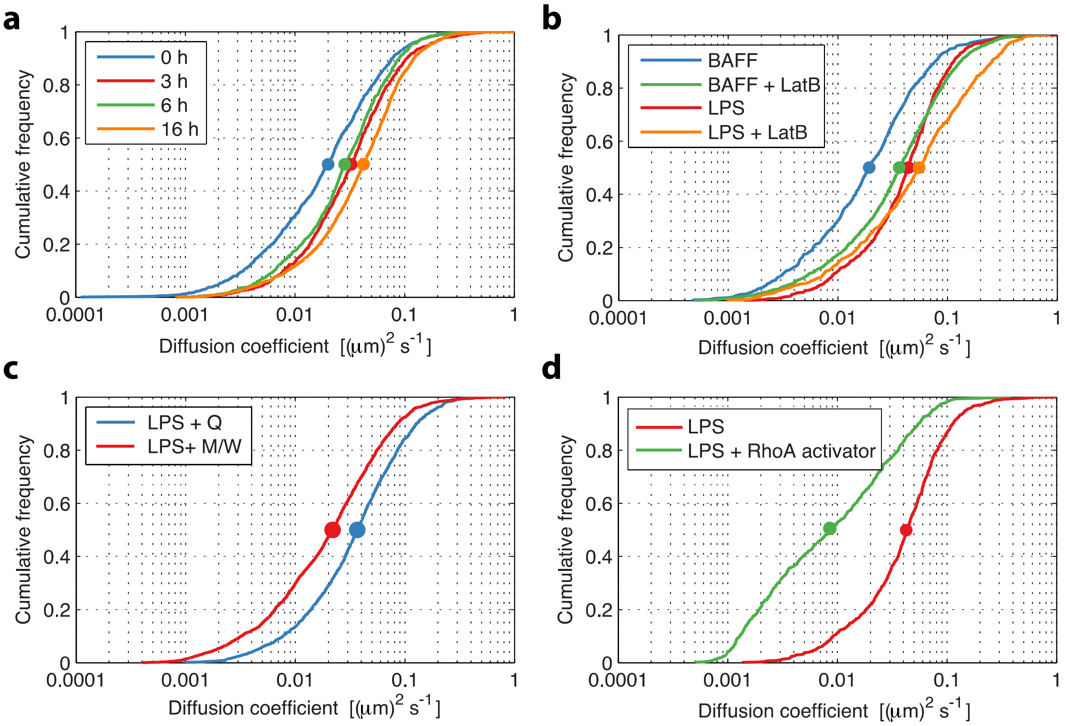


**Supplementary Figure 5 | Single-state analysis of BCR diffusion.** Primary B cells were treated as described below, labeled with biotinylated anti-IgM Fab fragments plus streptavidin-655 nm Qdots, and adhered to anti-MHCII-coated coverslips before performing SPT of BCRs on the ventral surface of the cells. Trajectories of individual mIgM-containing BCRs were generated from live-imaging videos taken at 33 frames per $s^{-1}$. Single-state diffusion coefficients (D) for individual tracks were calculated using a maximum likelihood estimation approach developed by Das *et al*.[4], as described in the Methods. The data are presented as cumulative frequency curves and the median values are indicted by the dots on the curves. For each condition, >500 tracks were analyzed.

(**a**) B cells were cultured overnight in 5 ng $ml^{-1}$ BAFF, with 5 μg $ml^{-1}$ LPS being added for the last 0-16 h of culture. These samples are identical to those for which MSS analysis is shown in **Fig. 2h-j** and HMM analysis is shown in **Supplementary Table 1**.

(**b**) B cells that had been cultured overnight with either BAFF or BAFF + 5 μg $ml^{-1}$ LPS were treated with DMSO or 1 μM latrunculin B (LatB) for 3 min. These samples are identical to those for which MSS analysis is shown in **Fig. 3** and HMM analysis is shown in **Supplementary Table 1**.

(**c**) B cells that had been cultured overnight with either BAFF + 5 μg $ml^{-1}$ LPS were treated with 5 μM of the M/W cofilin-blocking peptides or the control Q peptide for 5 min at 37°C. These samples are identical to those for which MSS analysis is shown in **Fig. 5b-d** and HMM analysis is shown in **Supplementary Table 1**.

(**d**) B cells that had been cultured overnight with BAFF + 5 μg $ml^{-1}$ LPS were incubated with or without the RhoA-activating peptide for 2-3 h. These samples are identical to those for which MSS analysis is shown in **Fig. 5h** and HMM analysis is shown in **Supplementary Table 1**.

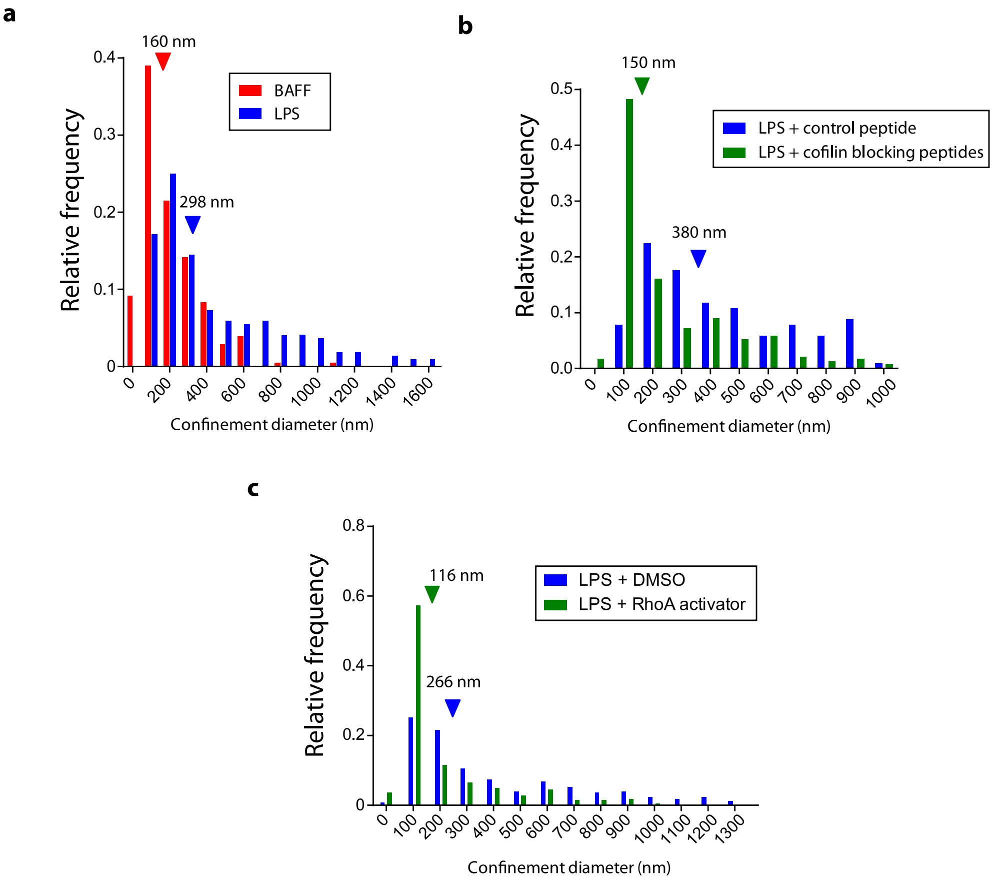


**Supplementary Figure 6 | Representative distributions of BCR confinement diameters.**

SPT of mIgM-containing BCRs was performed as in **Fig. 2**. Confinement diameters were determined for individual IgM-containing BCRs that were classified as exhibiting confined/sub-diffusive motion by the MSS algorithms. Confinement diameter values for individual BCRs were sorted into bins that are centered around multiples of 100 nm, e.g. 100 $\pm$ 50 nm.  The graphs display the relative frequency of BCR confinement diameters in all of the videos in which cells in a specific treatment group were analyzed within a single experiment. The total number of BCR trajectories per condition was >600 and the median confinement diameter values are indicated by the triangles.

Confinement diameter distributions are shown for mIgM-containing BCRs on:

(**a**) B cells that had been cultured overnight with either 5 ng ml$^{-1}$ BAFF or BAFF + 5 µg ml$^{-1}$ LPS (see **Supplementary Movies 1 and 2**, respectively, and **Fig. 2e**)

(**b**) B cells that had been cultured overnight with BAFF + 5 µg ml$^{-1}$ LPS and then treated with the M/W cofilin-blocking peptides or the control Q peptide for 5 min (see **Supplementary Movie 6** and **Fig. 5b-d**)

(**c**) B cells that had been cultured overnight with BAFF + 5 µg ml$^{-1}$ LPS and then treated with either DMSO or 1 µg ml$^{-1}$ of the RhoA activator for 2-3 h (see **Supplementary Movie 7** and **Fig. 5h**).

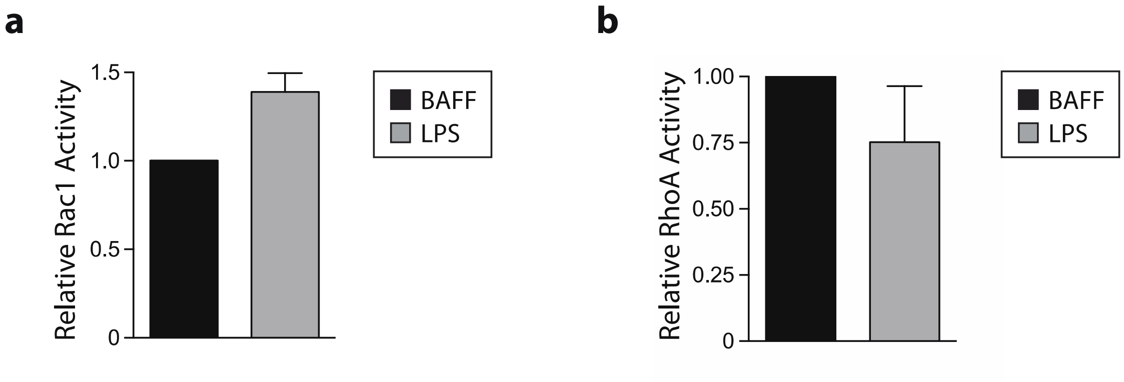


**Supplementary Figure 7 | Culturing B cells with LPS causes modest changes in the levels of active Rac1 and RhoA.** Primary B cells were cultured overnight with 5 ng $ml^{-1}$ BAFF or BAFF + 5 µg $ml^{-1}$ LPS. The amounts of active GTP-bound Rac1 (**a**) or RhoA (**b**) in cell extracts were determined using G-LISA assays. The data (mean ± s.e.m. for 3 independent experiments) are expressed relative to the amount of activated Rac1 or RhoA in extracts of BAFF-cultured cells.

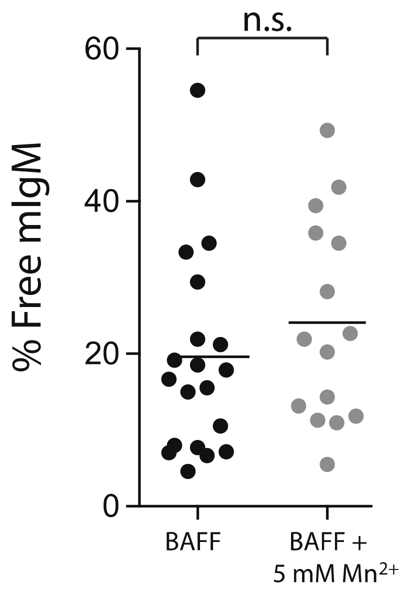


**Supplementary Figure 8 | Integrin activation does not increase BCR mobility.** Primary B cells were cultured overnight with 5 ng ml$^{-1}$ BAFF and then labeled with biotinylated anti-IgM Fab fragments plus streptavidin-655 nm Qdots. The cells were then incubated with or without 5 mM $Mn^{2+}$ for 20 min prior to imaging. Trajectories of individual mIgM-containing BCRs were generated as in **Fig. 2** and MSS analysis was used to classify trajectories of individual mIgM-containing BCRs as either confined or free. Each dot represents the percent of BCRs exhibiting free diffusion in a single video. Bars are mean values. n>15 videos per condition; n.s., not significant using Student's 2-tailed unpaired t-test.

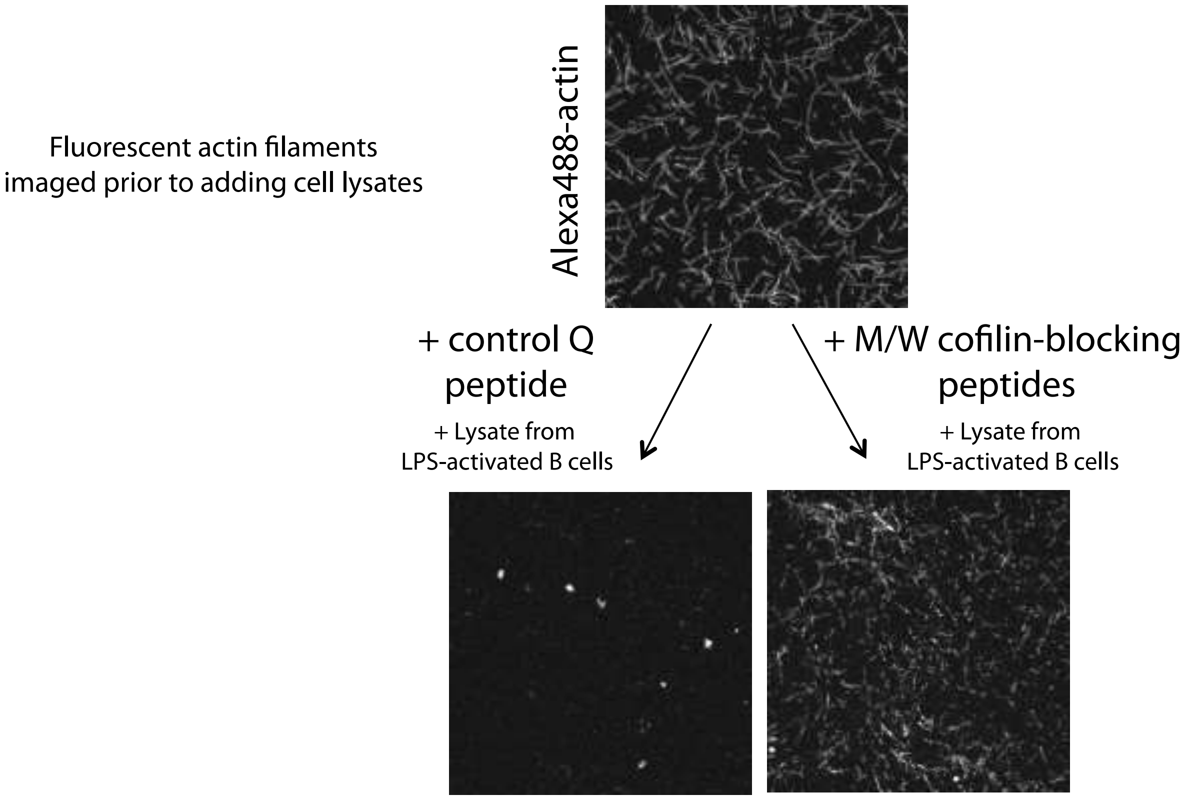


**Supplementary Figure 9 | Cofilin-blocking peptides inhibit the *in vitro* severing of actin filaments by cell extracts.** Actin filaments containing Alexa488-actin monomers and biotinylated actin monomers were adhered to slides coated with anti-biotin antibodies and then imaged. The control Q peptide or the M/W cofilin-blocking peptides were then allowed to bind to the immobilized actin filaments before washing the slides, and adding lysates from LPS-activated primary B cells. Substantial actin severing, as indicated by the decrease in fluorescent filaments that remained attached to the slide, occurred in the presence of the control Q peptide whereas the M/W cofilin-blocking peptides greatly reduced F-actin severing.

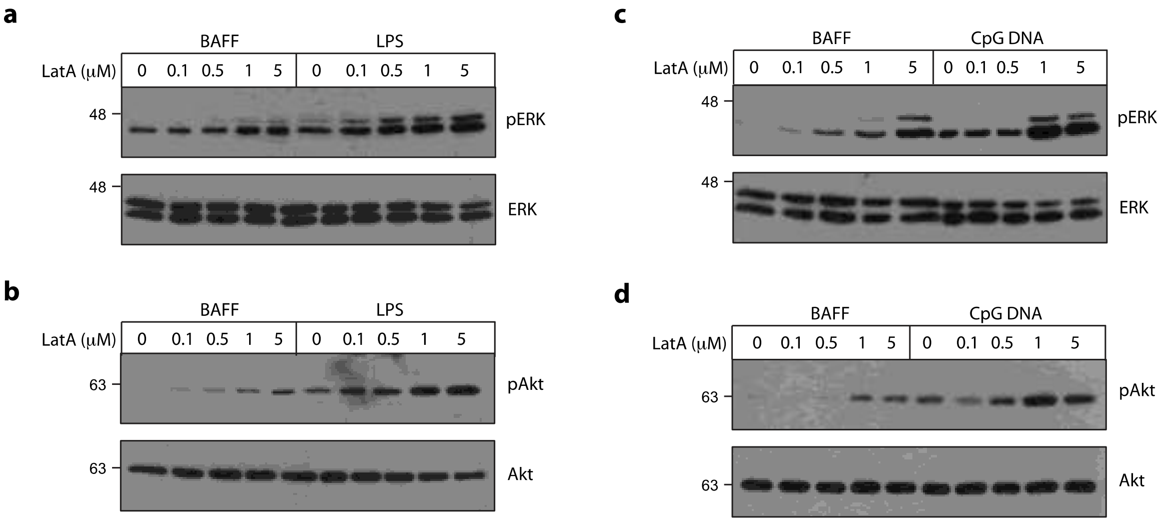


**Supplementary Figure 10 | Disrupting the actin cytoskeleton increases antigen-independent phosphorylation of ERK and Akt.** B cells were cultured overnight with 5 ng ml$^{-1}$ BAFF, BAFF + 5 µg ml$^{-1}$ LPS (**a,b)**, or BAFF + 0.5 µg ml$^{-1}$ CpG DNA (**c,d**) and then treated with DMSO (0) or the indicated concentrations of latrunculin A (LatA) for 5 min. Cell extracts were analyzed by immunoblotting with anti-phospho-ERK (pERK) and then anti-ERK (**a,c)** or with anti-phospho-Akt (pAkt) and then anti-Akt (**b,d)**. Comparing the DMSO (0) samples for BAFF-cultured B cells versus TLR-activated B cells shows that overnight exposure to TLR ligands increased tonic signalling. LatA caused further increases pERK and Akt in both resting and TLR-activated B cells. Data from these blots are graphed in **Fig. 7d**.

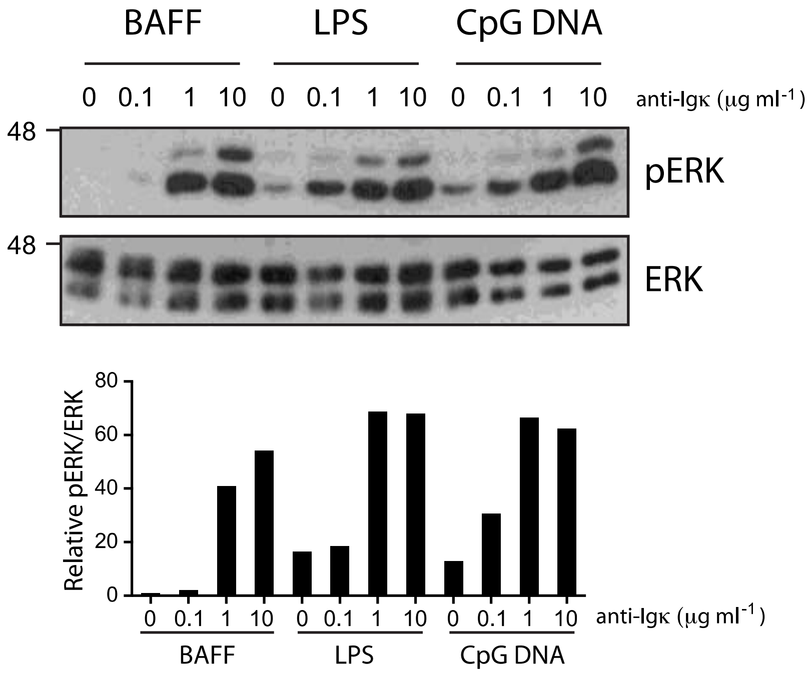


**Supplementary Figure 11 | Comparison of ERK phosphorylation induced by overnight priming with TLR ligands versus acute stimulation with anti-Igκ.** Primary B cells were cultured overnight with 5 ng ml$^{-1}$ BAFF, BAFF + 5 μg ml$^{-1}$ LPS, or BAFF + 0.5 μg ml$^{-1}$ CpG DNA before being stimulated for 5 min with the indicated concentrations of soluble anti-Igκ antibodies. Cell extracts were analyzed by immunoblotting with anti-pERK and then anti-ERK. A representative blot is shown. The graph shows the ratio of pERK/ERK relative to that in unstimulated BAFF-cultured B cells (means values for 3 experiments).

Comparing the 0 anti-Igκ lanes shows the increased basal pERK levels caused by overnight culture with LPS or CpG DNA. The anti-Igκ stimulation lanes show that TLR priming enhanced BCR-induced ERK phosphorylation at low concentrations (0.1 μg ml$^{-1}$) of anti-Igκ and that the increased basal level of pERK caused by TLR priming is less than that caused by acute stimulation of BAFF-cultured B cells with 1-10 μg ml$^{-1}$ anti-Igκ.

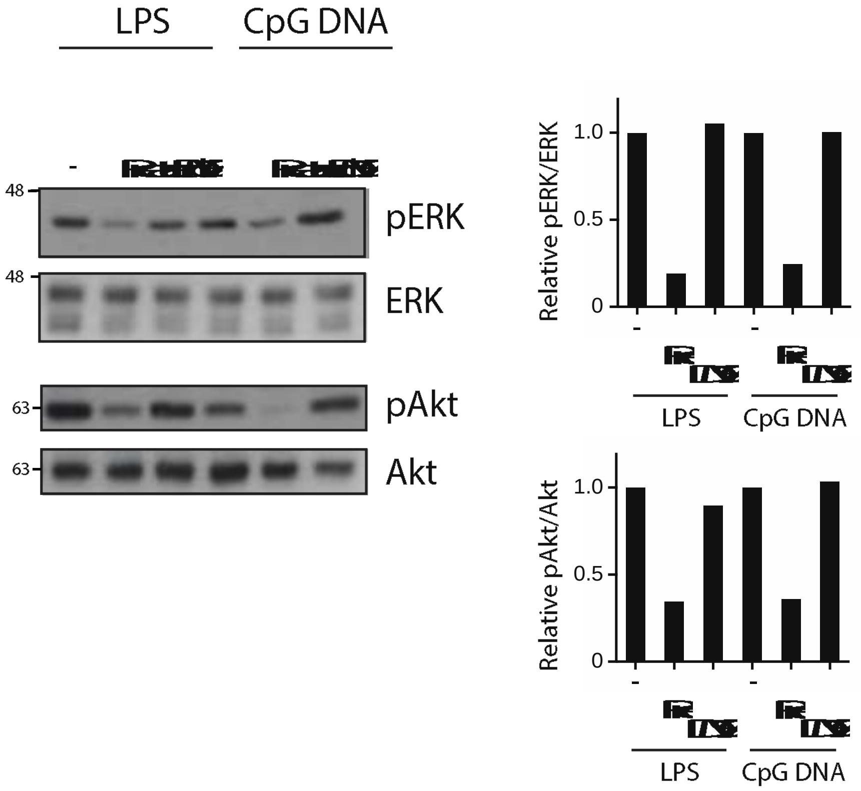


**Supplementary Figure 12 | The Syk inhibitor piceatannol decreases the levels of pERK and pAkt in TLR-primed cells.** B cells were cultured overnight with BAFF + 5 µg $ml^{-1}$ LPS, or BAFF + 0.5 µg $ml^{-1}$ CpG DNA. The cells were then treated with nothing (-), 10 µM piceatannol, or DMSO (vehicle control) for 30 min at 37$^{o}$C before being lysed. The levels of pERK, total ERK, pAkt, and total Akt in cell extracts were assessed by immunoblotting. The graphs show the ratios of pERK/ERK or pAkt/Akt normalized to those in untreated LPS- or CpG DNA-cultured cells (- lanes). Each bar is the mean for 3 independent experiments.

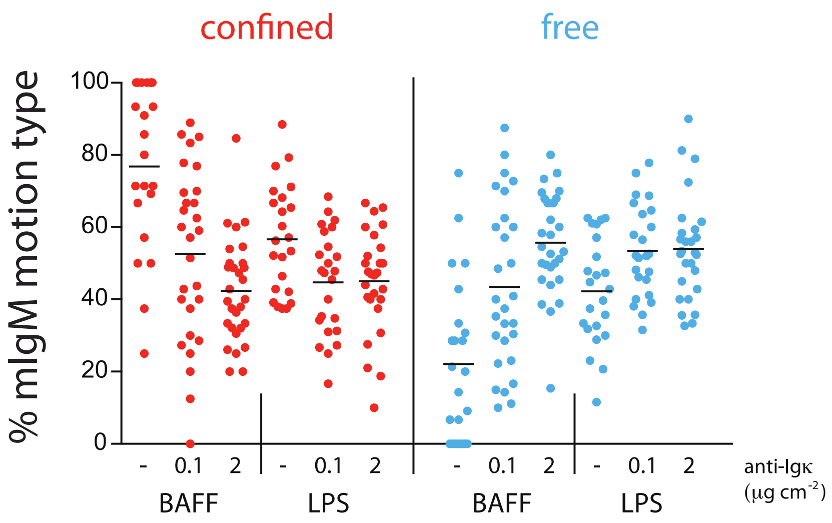


**Supplementary Figure 13 | TLR priming and acute BCR clustering combine to reduce the fraction of confined BCRs and increase the fraction of freely diffusing BCRs.** The SPT data from the samples in **Fig. 2a** (where single-state diffusion coefficients are presented) were analyzed using the MSS algorithms to determine the percent of confined versus free BCRs. B cells were cultured overnight with 5 ng $ml^{-1}$ BAFF or BAFF + LPS (5 μg $ml^{-1}$) and then labeled with biotinylated anti-IgM Fab fragments plus streptavidin Qdots. The cells were adhered for 5 min to coverslips coated with anti-MHCII (-) or the indicated densities of anti-Igκ SPT of non-engaged mIgM-containing BCRs on the dorsal surface of cells was performed. Each dot is the median value for >20 B cells from 3 experiments. Bars are mean values.

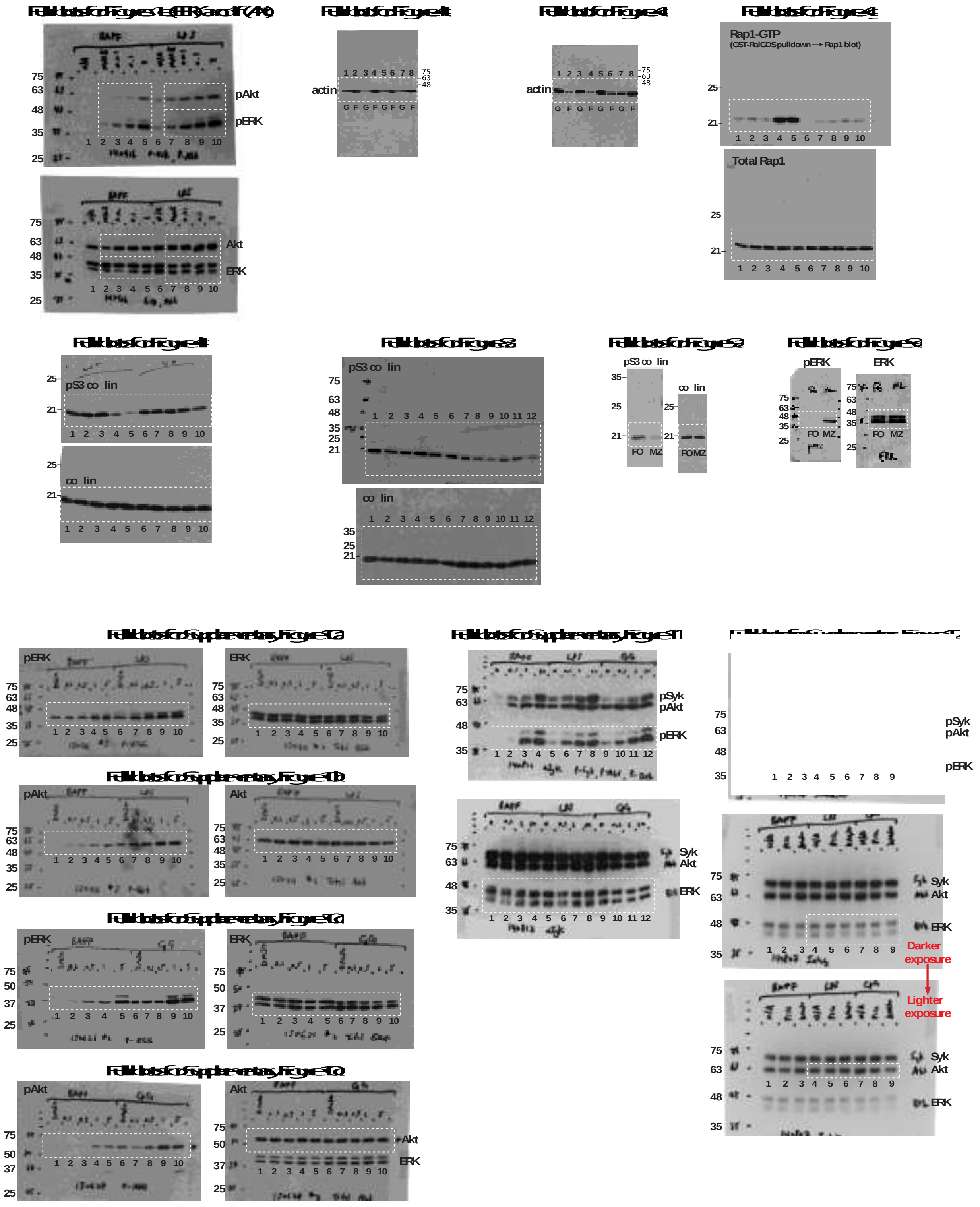


**Supplementary Figure 14 | Images of uncropped blots.** Images of full blots that were cropped for presentation in the figures are shown. The portions of the blots that appear in the figures are indicated by the white outlines. Molecular weight markers (in kDa) are on the sides of the blots. For each figure above, the samples in each lane of the blot are:

**Full blots for Figures 1e and 1f**: Primary splenic B cells were cultured overnight with 5 ng ml$^{-1}$ BAFF (lanes 1-5) or BAFF + 5 µg ml$^{-1}$ LPS (lanes 6-10). The cells were either kept in suspension (lanes 1 and 6; not used in Figs. 1e,f) or incubated for 10 min in wells coated with 2 µg cm$^{-1}$ anti-MHCII (lanes 2,7), 0.01 µg cm$^{-1}$ anti-Igκ (lanes 3,8), 0.1 µg cm$^{-1}$ anti-Igκ (lanes 4,9), or 2 µg cm$^{-1}$ anti-Igκ (lanes 5,10). Blots were probed with a mixture of pERK and pAkt antibodies (upper blot) or with a mixture of ERK and Akt antibodies (lower blot). Figure 1e shows pERK and ERK data. Figure 1f shows pAkt and Akt data.

**Full blot for Figure 4b**: Extracts were prepared from *ex vivo* primary B cells (lanes 1,2), B cells that had been cultured overnight with 5 ng ml$^{-1}$ BAFF (lanes 3,4), B cells that had been cultured overnight with BAFF + 5 µg ml$^{-1}$ LPS (lanes 5,6), or B cells that had been cultured overnight with BAFF + 0.5 µg ml$^{-1}$ CpG DNA (lanes 7,8). The cell extracts were then separated into soluble fractions containing G-actin (lanes labeled G) and insoluble fractions containing F-actin (lanes labeled F). Blots were probed with a β-actin antibody.

**Full blot for Figure 4c**: Primary splenic B cells were cultured overnight with 5 ng ml$^{-1}$ BAFF (lanes 1-4) or BAFF + 5 µg ml$^{-1}$ LPS (lanes 5,8). Cell extracts were sonicated to destroy existing actin filaments and either kept on ice (lanes 1,2 for BAFF-cultured cells; lanes 5,6 for LPS-cultured cells) or incubated for 10 min at 37$^{o}$C to allow *de novo in vitro* actin polymerization (lanes 3,4 for BAFF-cultured cells; lanes 7,8 for LPS-cultured cells). Samples were then separated into soluble fractions containing G-actin (odd-numbered lanes labeled G) and insoluble fractions containing F-actin (even-numbered lanes labeled F). Blots were probed with a β-actin antibody.

**Full blots for Figure 4g**: B cells were isolated from wild-type mice (lanes 1-5) or MyD88 knockout mice (lanes 6-10). Extracts were prepared from *ex vivo* B cells (lanes 1,6), B cells that had been cultured overnight with 5 ng ml$^{-1}$ IL-4 (lanes 2,7), B cells that had been cultured overnight with 5 ng ml$^{-1}$ BAFF (lanes 3,8), B cells that had been cultured overnight with BAFF + 5 µg ml$^{-1}$ LPS (lanes 4,9), or B cells that had been cultured overnight with BAFF + 0.5 µg ml$^{-1}$ CpG DNA (lanes 5,10). Cell extracts were probed for total Rap1 (lower blot) or assayed for levels of activated Rap1 (upper blot). Blots were probed with a Rap1 antibody.

**Full blots for Figure 4h**: B cells were isolated from wild-type mice (lanes 1-5) or MyD88 knockout mice (lanes 6-10). Extracts were prepared from *ex vivo* B cells (lanes 1,6), B cells that had been cultured overnight with 5 ng ml$^{-1}$ IL-4 (lanes 2,7), B cells that had been cultured overnight with 5 ng ml$^{-1}$ BAFF (lanes 3,8), B cells that had been cultured overnight with BAFF + 5 µg ml$^{-1}$ LPS (lanes 4,9), or B cells that had been cultured overnight with BAFF + 0.5 µg ml$^{-1}$ CpG DNA (lanes 5,10). Cell extracts were probed for cofilin that is phosphorylated on serine 3 (pS3 cofilin, upper blot) or total cofilin (lower blot).

**Full blots for Figure 8a**: *Ex vivo* B cells (lanes 1-3), as well as B cells that had been cultured overnight with 5 ng ml$^{-1}$ BAFF (lanes 4-6), BAFF + 5 µg ml$^{-1}$ LPS (lanes 7-9) or LPS alone (lanes 10-12), were left unstimulated (lanes 1,4,7,10) or stimulated with 1 µg ml$^{-1}$ anti-Igκ for 5 min (lanes 2,5,8,11) or 15 min (lanes 3,6,9,12). Cell extracts were probed for pS3 cofilin (upper blot) or total cofilin (lower blot).

**Full blots for Figure 9a**: Cell extracts from *ex vivo* follicular (FO) or marginal zone (MZ) B cells were probed for pS3 cofilin (left blot) or total cofilin (right blot).

**Full blots for Figure 9c**: Cell extracts from *ex vivo* FO or MZ B cells were probed for pERK (left blot) or total ERK (right blot).

**Full blots for Supplementary Figures 10a-d**: B cells were cultured overnight with 5 ng $ml^{-1}$ BAFF (lanes 1-5), BAFF + 5 µg $ml^{-1}$ LPS (lanes 6-10 in Supplementary Figs. 10a and 10b), or BAFF + 0.5 µg $ml^{-1}$ CpG DNA (lanes 6-10 in Supplementary Figs. 10c and 10d). The cells were then treated with DMSO (lanes 1,6), 0.1 µM latrunculin A (LatA; lanes 2,7), 0.5 µM LatA (lanes 3-8), 1 µM LatA (lanes 4,9) or 5 µM LatA (lanes 5,10) for 5 min. Cell extracts were probed for pERK and ERK (left and right blots, respectively in Supplementary Figs. 10a and 10c) or pAkt and Akt (left and right blots, respectively in Supplementary Figs. 10b and 10d).

**Full blots for Supplementary Figure 11**: B cells were cultured overnight with 5 ng $ml^{-1}$ BAFF (lanes 1-4), BAFF + 5 µg $ml^{-1}$ LPS (lanes 5-8), or BAFF + 0.5 µg $ml^{-1}$ CpG DNA (lanes 9-12). The cells were then stimulated in suspension with 0 (lanes 1,5,9), 0.1 µg $ml^{-1}$ (lanes 2,6,10), 1 µg $ml^{-1}$ (lanes 3,7,11), or 10 µg $ml^{-1}$ (lanes 4,8,12) anti-Igκ for 5 min. Blots were probed with a mixture of pSyk, pAkt and pERK antibodies (upper blot) or with a mixture of Syk, Akt, and ERK antibodies (lower blot). Only the pERK and ERK data are shown in Supplementary Fig. 11.

**Full blots for Supplementary Figure 12**: B cells were cultured overnight with 5 ng $ml^{-1}$ BAFF (lanes 1-3; not used in the figure), BAFF + 5 µg $ml^{-1}$ LPS (lanes 4-6), or BAFF + 0.5 µg $ml^{-1}$ CpG DNA (lanes 7-9). The cells were then treated with nothing (N/A, lanes 1,4,7), 10 µM piceatannol (lanes 2,5,8), or DMSO (lanes 3,6,9) for 30 min. Blots were probed with a mixture of pSyk (not shown in Supplementary Fig. 12), pERK and pAkt antibodies (upper blot) or with a mixture of Syk (not shown in Supplementary Fig. 12), ERK and Akt antibodies (lower two blots). The darker exposure of the ERK/Akt blot (middle blot) was used for the total ERK blot shown in Supplementary Fig. 12. The lighter exposure of the same ERK/Akt blot (lower blot) was used for the total Akt blot shown in Supplementary Fig. 12.

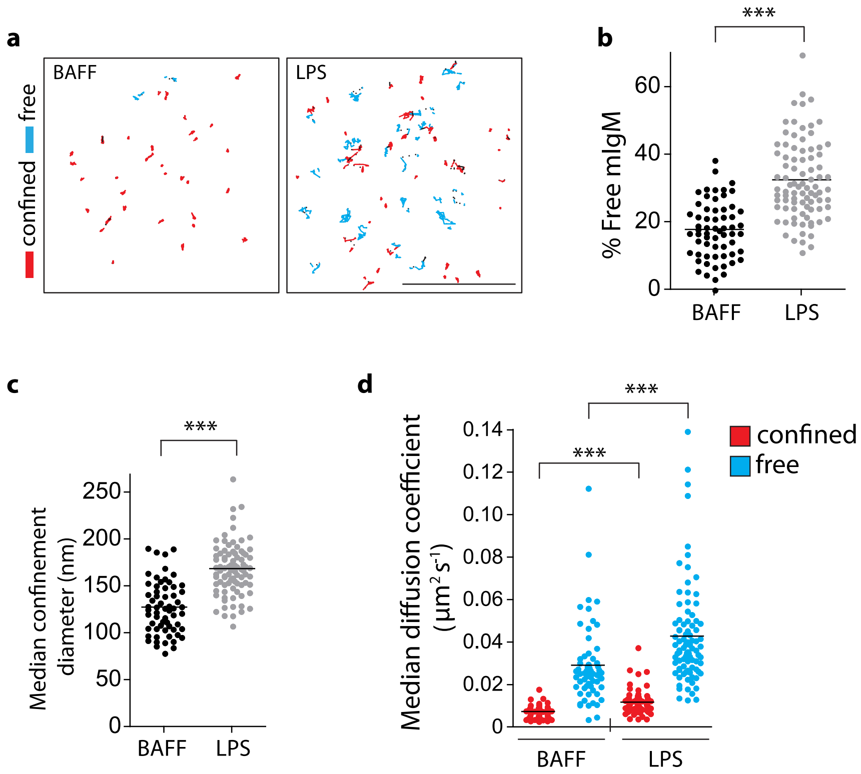


**Supplementary Figure 15 | TIRF imaging of Cy3-labeled BCRs.** B cells that were cultured overnight with 5 ng ml$^{-1}$ BAFF or BAFF + 5 µg ml$^{-1}$ LPS were labeled with 1-5 ng ml$^{-1}$ Cy3-anti-IgM Fab fragments and imaged by TIRF using an Olympus IX81 microscope equipped with a 150× oil-immersion objective (NA 1.45) and a Hamamatsu C9100-13 camera.

(**a**) Trajectories of individual mIgM-containing BCRs generated from live-imaging videos taken at 10 frames s$^{-1}$ for 10 s. Scale bar, 5 µm. Red, confined trajectories; cyan, free/unconfined trajectories; linear and undetermined trajectories (< 5% of total) are not shown.

(**b**) The percent of mIgM BCRs that exhibited free diffusion, as opposed to confined diffusion.

(**c**) Median confinement diameters for the confined mIgM BCRs

(**d**) Median diffusion coefficients for both confined and free mIgM-containing BCRs.

Each dot represents the trajectories from a single video. Horizontal lines are mean values for >50 videos per condition; >3000 trajectories per condition were analyzed. ***$P$ < 0.001, Student's unpaired 2-tailed t-test.

The mean positional accuracy for particles labeled with Cy3-anti-IgM Fab fragments was 17.8 nm (see **Supplementary Fig. 4**). The results obtained using this approach were similar to those obtained using biotinylated anti-IgM Fab fragments plus Qdots. Given the limited penetration depth of TIRF, these results confirm that only BCRs on the cell surface were tracked when we used Qdot labeling to visualize BCR trajectories.

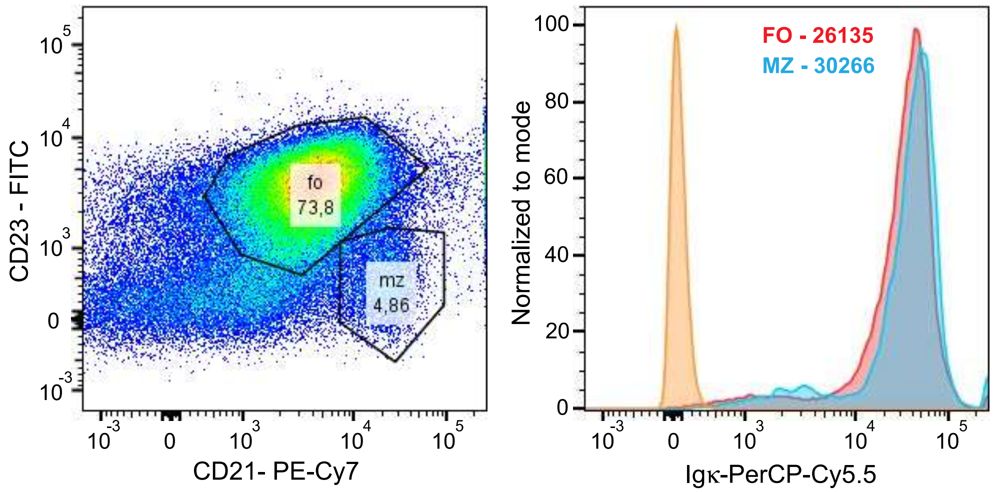


**Supplementary Figure 16 | FACS analysis of cell surface BCR levels on FO and MZ B cells.** Total splenic B cells were isolated by negative selection and then stained with anti-CD21-PE-Cy7, anti-CD23-FITC, and anti-Igκ-PerCP-Cy5.5. Igκ levels on FO versus MZ B cells (right panel) were determined by gating on $CD21^{int}CD23^{hi}$ (FO B cell) and $CD21^{hi}CD23^{lo}$ (MZ B cell) populations as shown in the left panel. In the right panel, the peak at the far left is for unstained cells. The numbers indicate the geometric mean fluorescence intensity. Similar results were obtained in 4 independent experiments.

| Condition | # of tracks analyzed | $D_{slow}$ ($10^{-3}$ µm$^2$ s$^{-1}$) | $D_{fast}$ ($10^{-2}$ µm$^2$ s$^{-1}$) | $k_{slow \rightarrow fast}$ (s$^{-1}$) | $k_{fast \rightarrow slow}$ (s$^{-1}$) | $K_{eff}$ = $k_{slow \rightarrow fast}$ / $k_{fast \rightarrow slow}$ |
|---|---|---|---|---|---|---|
| ***Fig. 2h-j*** | | | | | | |
| Control (BAFF only) | 1430 | **1.97** [1.94 – 2.00] | **4.66** [4.59 – 4.73] | **2.96** [2.87 – 3.06] | **3.86** [3.70 – 4.02] | **0.768** [0.739 – 0.797] |
| LPS, 3 h | 955 | **4.09** [3.96 – 4.22] | **6.49** [6.37 – 6.61] | **3.40** [3.26 – 3.56] | **3.19** [3.02 – 3.40] | **1.06** [1.01 – 1.13] |
| LPS, 6 h | 877 | **2.85** [2.78 – 2.93] | **5.12** [5.05 – 5.21] | **3.59** [3.45 – 3.74] | **2.88** [2.73 – 3.03] | **1.25** [1.19 – 1.31] |
| LPS, 16 h | 1902 | **5.29** [5.18 – 5.37] | **7.71** [7.61 – 7.79] | **2.56** [2.48 – 2.66] | **2.12** [2.03 – 2.22] | **1.21** [1.16 – 1.26] |
| ***Fig. 3*** | | | | | | |
| BAFF + DMSO | 808 | **2.18** [2.14 – 2.23] | **4.99** [4.89 – 5.09] | **3.01** [2.91 – 3.13] | **4.61** [4.38 – 4.85] | **0.653** [0.621 – 6.88] |
| BAFF + LatB | 1793 | **3.10** [3.04 – 3.15] | **7.03** [6.95 – 7.10] | **3.12** [3.02 – 3.23] | **2.45** [2.35 – 2.56] | **1.27** [1.23 – 1.32] |
| LPS + DMSO | 905 | **5.76** [5.62 – 5.95] | **7.07** [6.96 – 7.21] | **2.71** [2.55 – 2.84] | **1.90** [1.76 – 2.04] | **1.43** [1.33 – 1.52] |
| LPS + LatB | 1025 | **4.26** [4.14 – 4.38] | **12.2** [12.0 – 12.4] | **2.86** [2.71 – 2.99] | **2.02** [1.89 – 2.15] | **1.42** [1.33 – 1.49] |
| ***Fig. 5b-d*** | | | | | | |
| LPS + control Q peptide | 2163 | **5.27** [5.18 – 5.37] | **8.01** [7.91 - 8.11] | **2.47** [2.44 – 2.57] | **2.45** [2.34 – 2.60] | **1.01** [0.969 – 1.05] |
| LPS + M/W cofilin-blocking peptides | 1619 | **1.97** [1.94 – 1.99] | **4.65** [4.60 – 4.70] | **2.84** [2.77 – 2.89] | **3.40** [3.33 – 3.53] | **0.834** [0.798 – 0.854] |
| ***Fig. 5h*** | | | | | | |
| LPS | 569 | **4.85** [4.71 – 4.99] | **6.19** [6.07 – 6.32] | **1.96** [1.80 – 2.10] | **1.78** [1.62 – 1.94] | **1.10** [1.01 – 1.19] |
| LPS + RhoA activator | 738 | **1.63** [1.61 – 1.66] | **3.69** [3.62 – 3.76] | **1.19** [1.15 – 1.26] | **2.21** [2.11 – 2.38] | **0.540** [0.504 – 0.566] |
| | | | | | | |

**Supplementary Table 1 | Two-state hidden Markov model (HMM) analysis of BCR diffusion.** Primary B cells were treated as described below, labeled with biotinylated anti-IgM Fabs plus streptavidin Qdots, and adhered to anti-MHCII-coated coverslips before performing SPT of BCRs on the ventral surface of the cells. Trajectories of individual mIgM-containing BCRs were generated from live-imaging videos taken at 33 frames s$^{-1}$ and analyzed using a two-state HMM algorithm[4]. This model assumes that individual trajectories consist of slow and fast segments and calculates the distribution of diffusion coefficients for each state as well as the probability of transitions between the two states. Diffusion coefficients for the slow and fast states ($D_{slow}$, $D_{fast}$) are shown along with the frequency of transitions between the two states ($k_{slow \rightarrow fast}$, $k_{fast \rightarrow slow}$). $K_{eff}$, the ratio of $k_{slow \rightarrow fast}$ divided by $k_{fast \rightarrow slow}$, indicates whether the predominant mode of switching is to the fast state ($K_{eff}$ >1) or to the slow state ($K_{eff}$ <1). The ranges in parentheses indicate 95% confidence intervals. For each condition, >500 BCR trajectories were analyzed.

The samples analyzed using this HMM algorithm are the same as those in the following figures, which show the corresponding MSS analysis:

a) **Fig. 2h-j**. B cells were cultured overnight in 5 ng ml$^{-1}$ BAFF, with 5 µg ml$^{-1}$ LPS being added for the last 0-16 h of culture. Single-state analysis for these samples is shown in **Supplementary Fig. 5a**.

b) **Fig. 3**. B cells that had been cultured overnight with either BAFF or BAFF + LPS were treated with DMSO or 1 µM latrunculin B (LatB) for 3 min. Single-state analysis for these samples is shown in **Supplementary Fig. 5b**.

c) **Fig. 5b-d**. B cells that had been cultured overnight with BAFF + LPS were treated with 5 µM of the M/W cofilin-blocking peptides or the control Q peptide for 5 min. Single-state analysis for these samples is shown in **Supplementary Fig. 5c**.

d) **Fig. 5h**. B cells that had been cultured overnight with BAFF + LPS were incubated with or without the RhoA-activating peptide for 2-3 h. Single-state analysis for these samples is shown in **Supplementary Fig. 5d**.

| Condition | Confinement diameter mean ± s.e.m. (nm) |
|---|---|
| ***Fig. 2c-f*** | |
| *Ex vivo* | 129.8 ± 7.3 |
| BAFF | 148.7 ± 11.3 |
| LPS | 261.4 ± 23.8 |
| CpG DNA | 255.2 ± 21.2 |
| ***Fig. 2h-j*** | |
| Control | 124.0 ± 12.4 |
| LPS, 3 h | 219.6 ± 17.1 |
| LPS, 6 h | 211.6 ± 17.8 |
| LPS, 16 h | 276.0 ± 23.6 |
| ***Fig. 3*** | |
| BAFF + DMSO | 128.4 ± 16.7 |
| BAFF + LatB | 276.6 ± 33.9 |
| LPS + DMSO | 316.7 ± 14.3 |
| LPS + LatB | 387.1 ± 23.4 |
| ***Fig. 5b-d*** | |
| BAFF + control peptide | 178.7 ± 17.2 |
| BAFF + cofilin-blocking peptide | 179.8 ± 12.4 |
| LPS + control peptide | 226.8 ± 27.0 |
| LPS + cofilin-blocking peptide | 140.5 ± 8.5 |
| ***Fig. 5h*** | |
| LPS + DMSO | 228.3 ± 14.2 |
| LPS + RhoA activator | 142.6 ± 10.8 |
| | |
| LPS + 4% paraformaldehyde | 33.2 ± 3.7 |

**Supplementary Table 2 | Summary of changes in BCR confinement diameters.**

Primary B cells were treated as described below, labeled with biotinylated anti-IgM Fab fragments plus streptavidin-655 nm Qdots, and adhered to anti-MHCII-coated coverslips before performing SPT of BCRs on the ventral surface of the cells. The trajectories were analyzed using MSS analysis as described in the Methods. For BCRs classified as exhibiting confined/sub-diffusive motion, the MSS algorithms calculated the maximum diameter of the confinement region. The values in this table are the mean ± s.e.m. for median confinement diameters from >10 videos per condition; >500 trajectories were analyzed per condition.

The samples for which confinement diameters are reported above are the same as those in the following figures:

a) **Fig. 2h-j**. B cells were cultured overnight in 5 ng $ml^{-1}$ BAFF, with 5 µg $ml^{-1}$ LPS being added for the last 0-16 h of culture.

b) **Fig. 3**. B cells that had been cultured overnight with either BAFF or BAFF + 5 µg $ml^{-1}$ LPS were treated with DMSO or 1 µM latrunculin B (LatB) for 3 min.

c) **Fig. 5b-d**. B cells that had been cultured overnight with BAFF + 5 µg $ml^{-1}$ LPS were treated with 5 µM of the M/W cofilin-blocking peptides or the control Q peptide for 5 min.

d) **Fig. 5h**. B cells that had been cultured overnight with BAFF + 5 µg $ml^{-1}$ LPS were incubated with or without the RhoA-activating peptide for 2-3 h.

When B cells were fixed with 4% paraformaldehyde, BCRs were rendered immobile. The median confinement diameter calculated for BCRs on these fixed cells reflects the size of the Qdot (15-20 nm) as well as vibrational movement of the Qdot. This value provides an estimate of the accuracy of the confinement diameter measurements. For almost all of the conditions shown in this table, the confinement diameter value for immobile BCRs on fixed cells was <25% of the measured confinement diameter value. Changes in the confinement diameter values caused by various treatments were sufficiently large that this value for immobile BCRs does not affect the conclusions drawn from the data.

## Supplementary References


1. Lin, K.B. *et al.* The Rap GTPases regulate B cell morphology, immune-synapse formation, and signaling by particulate B cell receptor ligands. *Immunity* **28**, 75-87 (2008).
2. Freeman, S.A. *et al.* Cofilin-mediated F-actin severing is regulated by the Rap GTPase and controls the cytoskeletal dynamics that drive lymphocyte spreading and BCR microcluster formation. *J. Immunol*. **187**, 5887-5900 (2011).
3. Jaqaman, K. *et al.* Robust single-particle tracking in live-cell time-lapse sequences. *Nat. Methods* **5**, 695-702 (2008).
4. Das, R., Cairo, C.W. & Coombs, D. A hidden Markov model for single particle tracks quantifies dynamic interactions between LFA-1 and the actin cytoskeleton. *PLoS Comput. Biol*. **5**, e1000556 (2009).
